# Supplementary material for: Towards universal neural network potential for material discovery applicable to arbitrary combination of 45 elements
Source: Nat Commun. 2022 May 30;13:2991. doi: 10.1038/s41467-022-30687-9 (PMC9151783; doi:10.1038/s41467-022-30687-9)
Supplement: Supplementary file 1 — Supplementary Information [file 41467_2022_30687_MOESM1_ESM.pdf]

Supplementary Information: Towards Universal Neural Network Potential for Material Discovery Applicable to Arbitrary Combination of 45 Elements

S Takamoto et al.

NOTE 1: NN ARCHITECTURE BENCHMARK USING OC20 DATASET

As mentioned in the Introduction, the OC20 dataset targets adsorbed structures on crystal surfaces. Numerical experiments suggest that this is a more challenging task than molecular or crystal structure data. This dataset was used to evaluate the performance of the architecture of the PFP.

First, we demonstrate the performance of the PFP architecture on the structure of energy and forces (S2EF) task [1]. We used the S2EF 2M dataset as training data, which is a sub-dataset two orders of magnitude smaller than the largest dataset provided by OC20. For evaluation, we used the validation dataset, which was not used in the training process. The values of the baseline models (SchNet and DimeNet++) correspond to the test datasets. The results are listed in Supplementary Table 1. The PFP architecture showed a good performance compared with the baseline models. See Supplementary Note 4 for the details of the calculation conditions.

It should be emphasized that even though the OC20 dataset covers a wide range of adsorbed structures, the model trained using the OC20 dataset is insufficient for the material discovery task as defined in this study. As a demonstration, we calculated the energies and densities of various crystal structures of silicon using the PFP architecture trained using only the OC20 dataset, and DimeNet++, which has an excellent score in the existing OC20 leaderboard. The elements supported in the OC20 dataset are shown in Supplementary Note 8.

The results showed a similar trend for both models. The first problem is the estimation of a stable structure. Both the DimeNet++ and PFP architectures trained with OC20 failed to estimate that the diamond structure was the most stable in both architectures. The most stable structure is body-centered cubic (BCC) for DimeNet++ and face-centered cubic (FCC) for the PFP architecture trained with OC20. This inconsistency may not be a problem when simulating silicon during the diamond structure phase. However, this is a problem in the material exploration task, where the other structures are predicted to be more stable. The second problem can be observed in systems with large deformations. When plotting the energy surface against the volume change, another stable point was often found far from the original stable bond distance. See Supplementary Note 3 for the detailed results. These problems are thought to be due to the limited diversity of structures covered by the dataset. The results indicate that, to build a universally applicable NNP for material discovery, we need to pay attention to the variety of structures in the dataset.

Model	ID	OOD Ads	OOD Cat	OOD Both
	Energy mean absolute error (MAE) [eV] ($\downarrow$)			
SchNet	0.4426	0.4907	0.5288	0.7161
DimeNet++	0.4858	0.4702	0.5331	0.6482
PFP (OC20)	0.2258	0.2345	0.4044	0.4762
	Force MAE [eV/Å] ($\downarrow$)			
SchNet	0.0493	0.0527	0.0508	0.0652
DimeNet++	0.0443	0.0458	0.0444	0.0558
PFP (OC20)	0.0418	0.0453	0.0455	0.0534
	Force cosine ($\uparrow$)			
SchNet	0.3180	0.2960	0.2943	0.3001
DimeNet++	0.3623	0.3470	0.3462	0.3685
PFP (OC20)	0.4848	0.4743	0.4559	0.4888
	EFwT ($\uparrow$)			
SchNet	0.11%	0.06%	0.07%	0.01%
DimeNet++	0.10%	0.03%	0.05%	0.01%
PFP (OC20)	0.02%	0.00%	0.00%	0.00%

Supplementary Table 1. Open Catalyst 2020 S2EF task. ID and OOD refer to the in-domain and out-of-domain datasets, respectively. SchNet and DimeNet++ were extracted from the leaderboard. For the PFP, the validation dataset was used instead. See the original reference [1] for the definition of the tasks.

NOTE 2: PFP REGRESSION BENCHMARKS FOR OUR DATASET

For the regression benchmark, we extracted three types of components from PFP dataset. See Supplementary Note 10 for the details of PFP dataset creation method.

The first type consists of disordered structures. The structure generation process is as follows: First, atoms are randomly selected from the periodic table and placed in the simulation cell. Next, the system is melted at approximately 10000 K through an MD simulation. An additional MD simulation is then conducted at 2000 K. See Supplementary Note 10 (disordered section) for details. The typical number of atoms in a single structure is 32. The structures created in this manner are expected to cover a vast range of phase spaces with little dependence on prior knowledge. This dataset is expected to provide a highly stringent assessment of the universality of the model. The structures produced in this fashion are a class of the most challenging configurations for prediction because of their highly disordered nature. In fact, structures encountered in practical problems are generally much more stable than disordered structures in terms of energy.

The second type consists of the adsorbed structures. It consists of a crystal surface and small molecules that are sufficiently close to interact.

The third type consists of molecules generated through normal-mode sampling (NMS). Specifically, this refers to the structures of organic molecules containing eight heavy atoms (including C, N, O, P, and S) whose atomic positions fluctuate according to the NMS method.[2]

Supplementary Table 2 shows the prediction performances of the energy and force for these components. For the last two realistic components, we can see that the PFP can predict energy and force with high accuracy. Structures used in this section are not used for the training process.

The detailed scatter plots corresponding to this section is available in Supplementary Note 5.

Lattice	Energy MAE [meV/atom]	Force MAE [eV/Å]
Disordered structure	13.6	0.13
Adsorbed structure	5.6	0.065
Molecule NMS structure (Molecule mode)	2.6	0.034

Supplementary Table 2. Energy and force regression performance among the datasets.

NOTE 3: ESTIMATED SILICON CRYSTAL PROPERTIES USING NNPS TRAINED USING OC20 DATASET

Supplementary Table 3 shows the estimated relative energies and densities of silicon crystals. DimeNet++ and PFP (OC20) are trained using the OC20 dataset. For comparison, PFP trained by our dataset is also shown in the PFP (ours) column. It should be noted that our dataset contains such crystal structures, and therefore the high accuracy of the results of PFP (ours) was expected.

The calculated wide energy surfaces are shown in Supplementary Figures 1, 2, and 3.

Property	Lattice	DFT	DimeNet++	PFP (OC20)	PFP (ours)
Relative energy	Diamond	(−4.56)			
[eV/atom]	FCC	0.54	−0.07	−2.36	0.43
	HCP	0.49	−0.26	−4.09	0.43
	BCC	0.55	−0.28	−0.85	0.48
	SC	0.32	−0.02	0.26	0.29
	Graphene	0.66	−0.25	0.58	0.59
Density	Diamond	2.28	2.09	2.28	2.28
[g/cm^3]	FCC	3.27	2.92	2.68	3.23
	HCP	3.25	2.99	2.66	3.25
	BCC	3.17	3.56	2.60	3.21
	SC	2.87	2.58	2.71	2.87

Supplementary Table 3. Comparison of estimated relative energies (compared to diamond structure) and densities of silicon crystals.

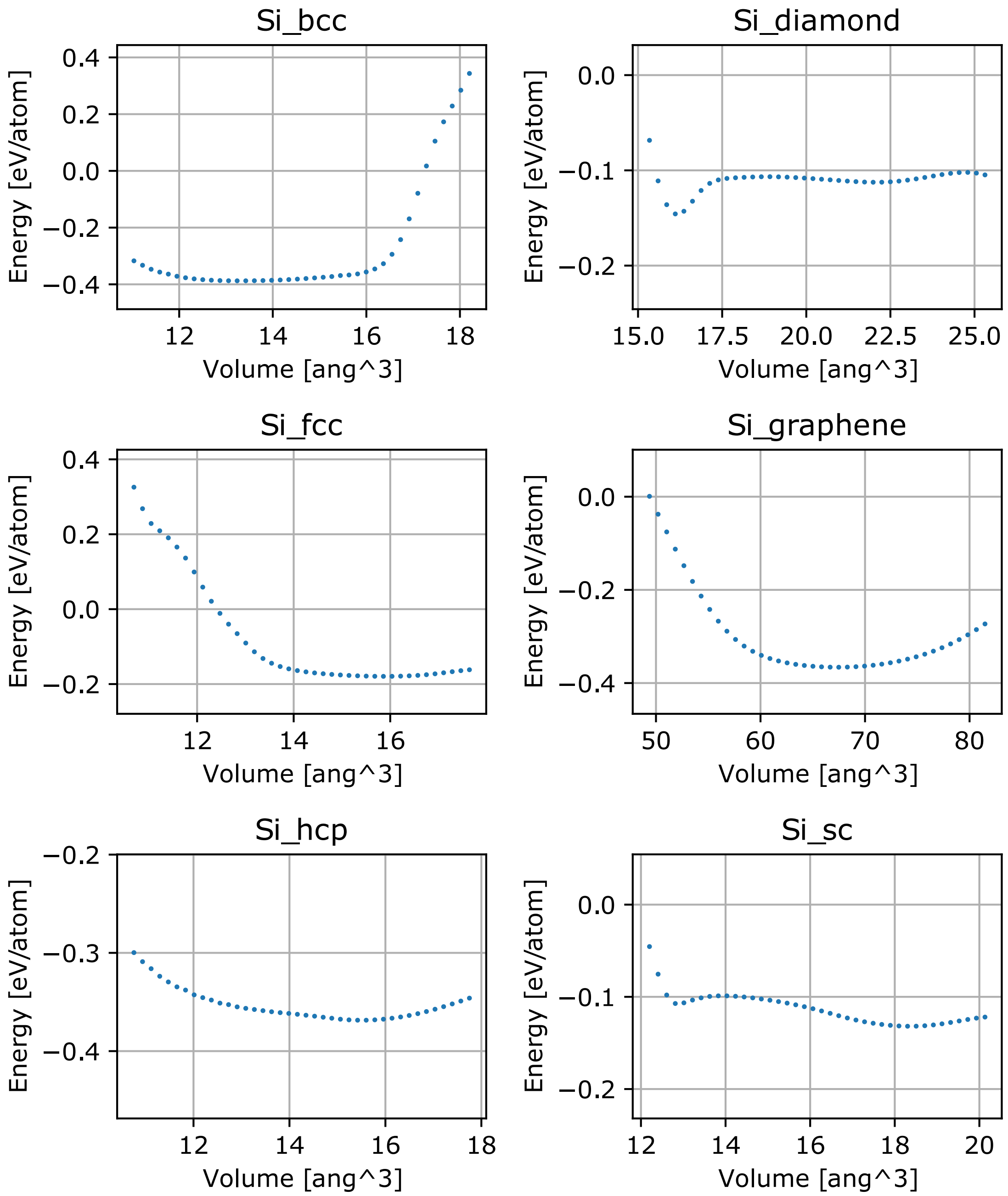

Supplementary Figure 1. Energy curve of DimeNet++. [1]

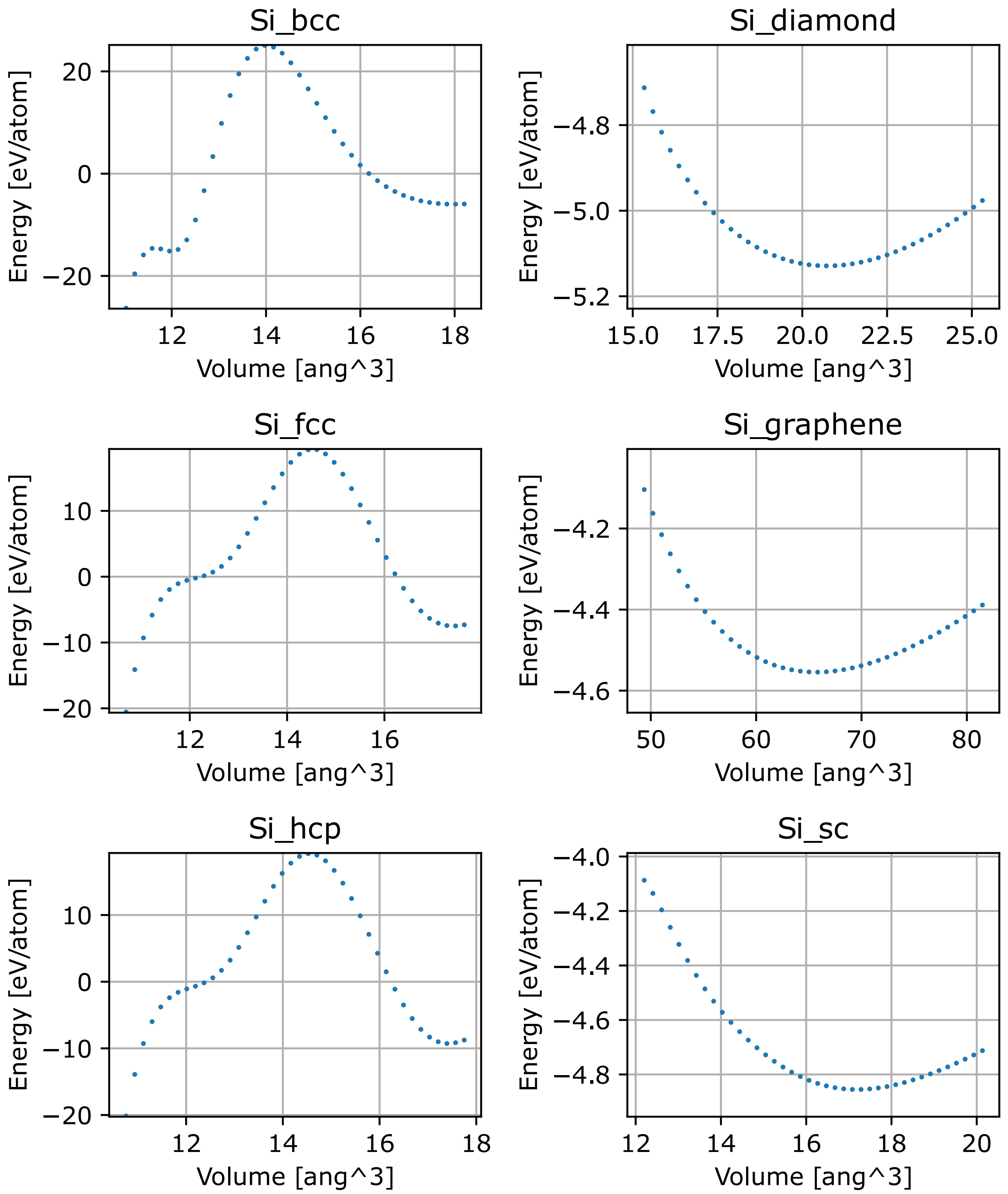

Supplementary Figure 2. Energy curve of PFP architecture trained with OC20 dataset only.

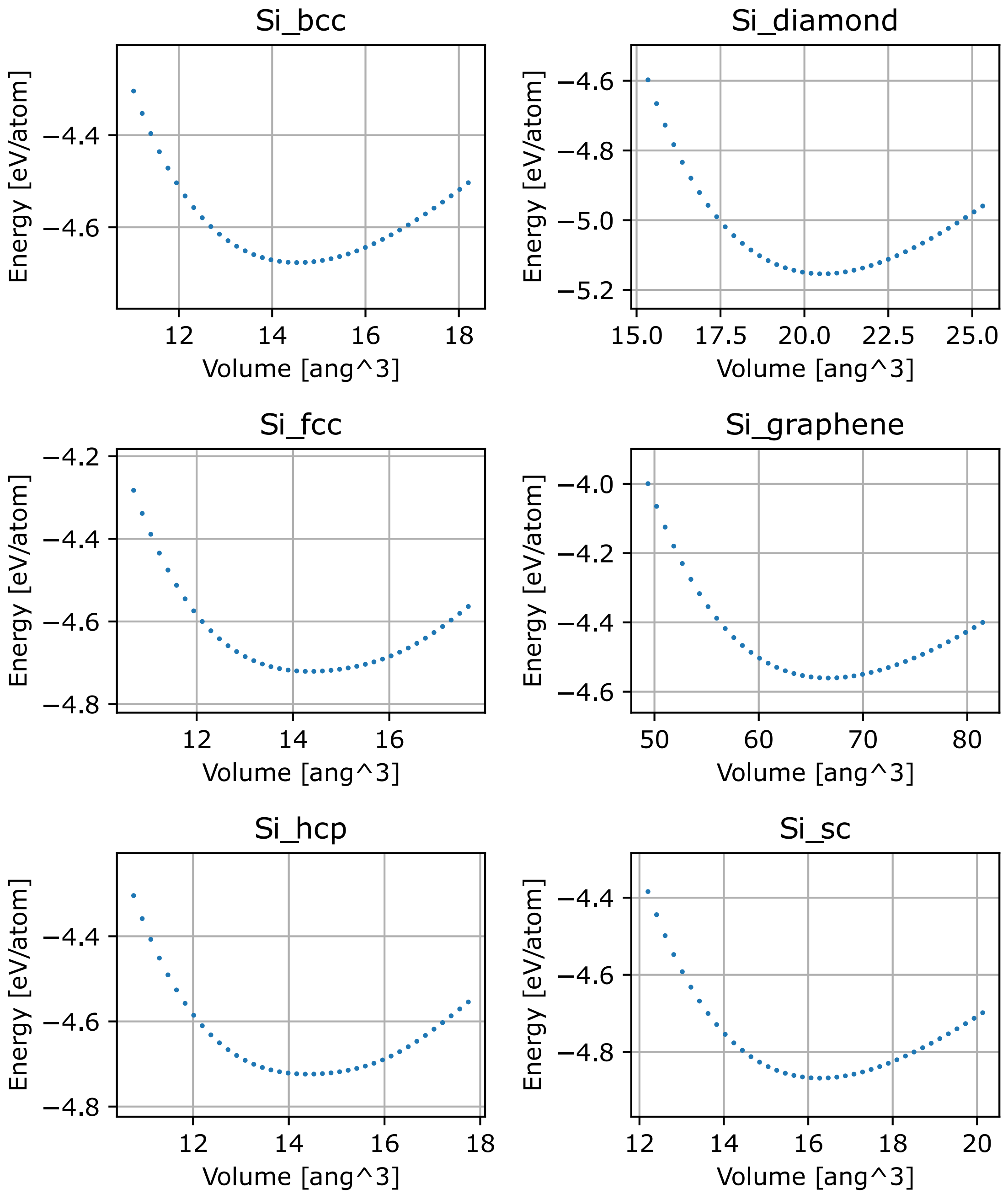

Supplementary Figure 3. Energy curve of ordinary PFP (trained using our dataset).

NOTE 4: PFP ARCHITECTURE FOR OC20 TASK

For the OC20 task, we essentially used the same architecture as the original PFP. However, the following points have been modified from the original: The NN parameters derived from the PFP dataset and the corresponding DFT calculations were excluded. This includes a shift in the value of the energy in a vacuum for each element. Training was conducted using only the OC20 S2EF 2M dataset, and no validation dataset was used during training. The validation dataset contains one million structures for each task. During inference, energy was clipped to a maximum of 10.0 eV/atom, and the force was clipped to a maximum of 100.0 eV/Å. There were three, four, three, and seven samples that met the conditions for the ID, OOD ads, OOD cat, and OOD for both tasks, respectively.

NOTE 5: DATASET REGRESSION PERFORMANCE

Supplementary Figure 4 shows a scatter plot of the regression results for energy and force in the sub components of the PFP dataset. They correspond to test dataset, which were randomly extracted from the dataset and not used during the training process. See Supplementary Note 10 for the description of each components.

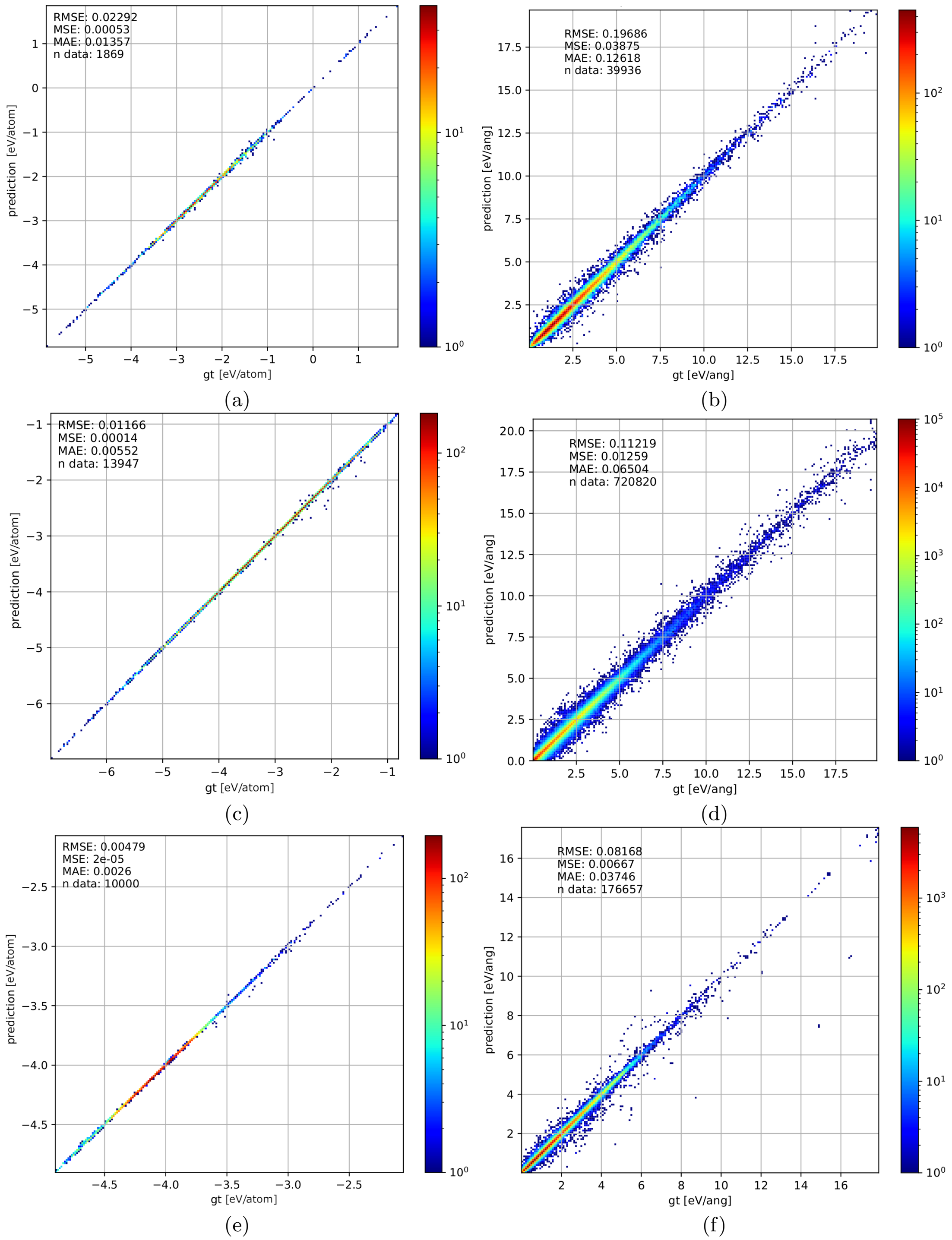

Supplementary Figure 4. Energy and force regression performance. The left column corresponds to energy (eV/atom), and the right column corresponds to the force (eV/Å). (a), (b) Disordered structures. (c), (d) Surface adsorbed structures. (e), (f): PubChem molecule normal-mode sampling (NMS) structures.

NOTE 6: CALCULATION TIME BENCHMARK

The energy and force calculation time for a system of 3000 Pt atoms using PFP was 0.3 s. By contrast, the estimated typical DFT calculation time for the same system is approximately 2 months, which means that PFP is 20-million times faster than DFT.

To estimate the DFT calculation time, we used QUANTUM ESPRESSO[3, 4] version 6.4.1. The pseudopotential applied for the calculation was Pt.pbe-n-kjpaw_psl.1.0.0.UPF from http://www.quantum-espresso.org, and the cutoff energy was set to 40 Ry. The calculation time was measured on an Intel Xeon Gold 6254 3.1 GHz×2 (36-core) CPU. The measured structures were bulk FCC platinum systems with 32, 108, and 256 atoms, and the calculation times were 34, 811, and 8280 s, respectively. We extrapolated the time required for the structure of 3000 atoms by fitting these values to a one-log graph. The fitted line shows that the calculation time is proportional to $O\left(N^{2.64}\right)$, where N is the number of atoms.

The PFP calculation time was measured on an NVIDIA V100 (single GPU).

NOTE 7: STRUCTURE EXAMPLES OF PFP DATASET

Supplementary Figure 5 shows an example of a structure in the PFP dataset.

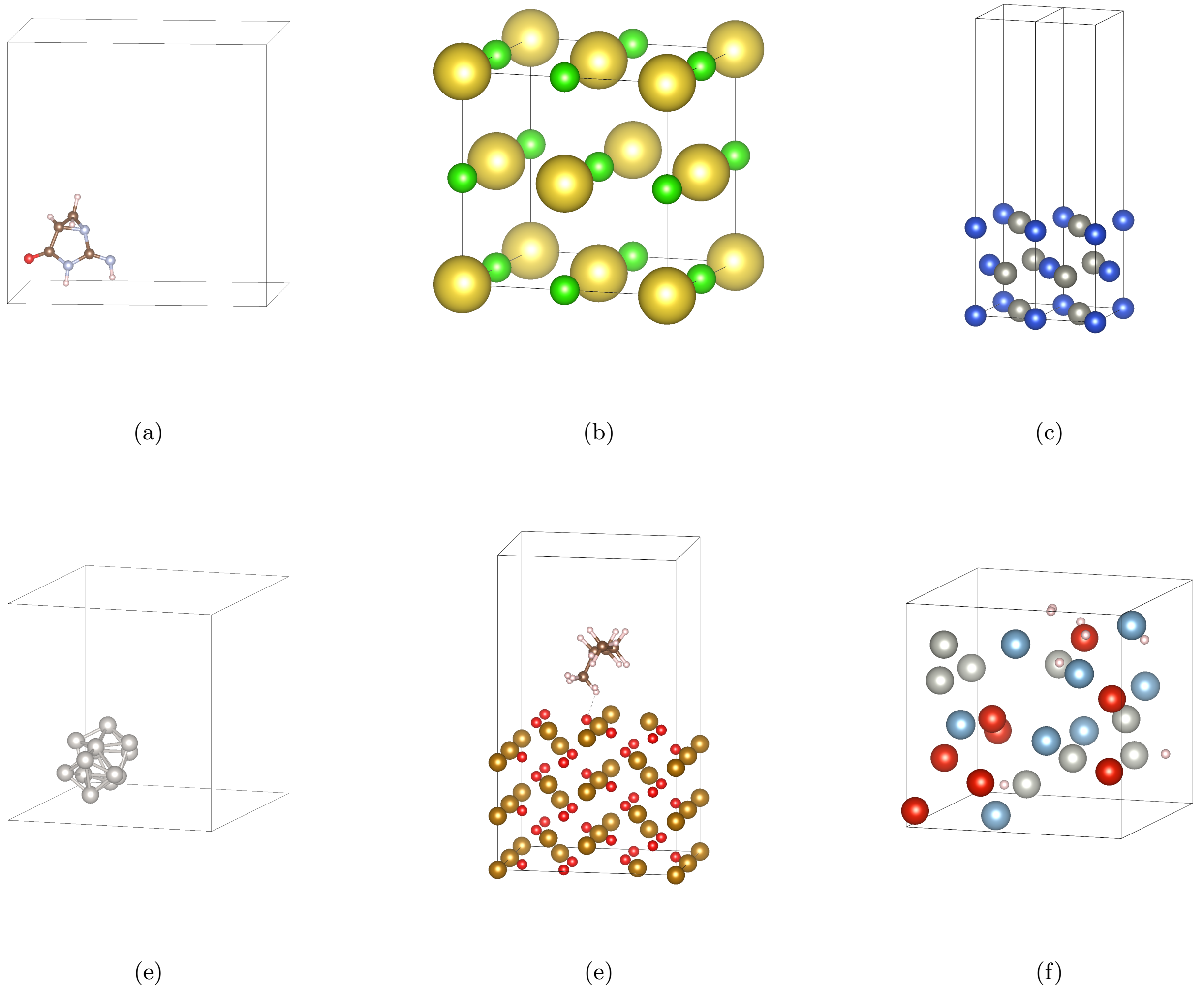

Supplementary Figure 5. Structural examples of PFP dataset: (a) molecule, (b) bulk, (c) slab, (d) cluster, (e) adsorption, and (f) disordered are shown.

NOTE 8: ELEMENTS SUPPORTED IN THE OC20 DATASET

Supplementary Figure 6 shows the 56 elements supported by the OC20 dataset.

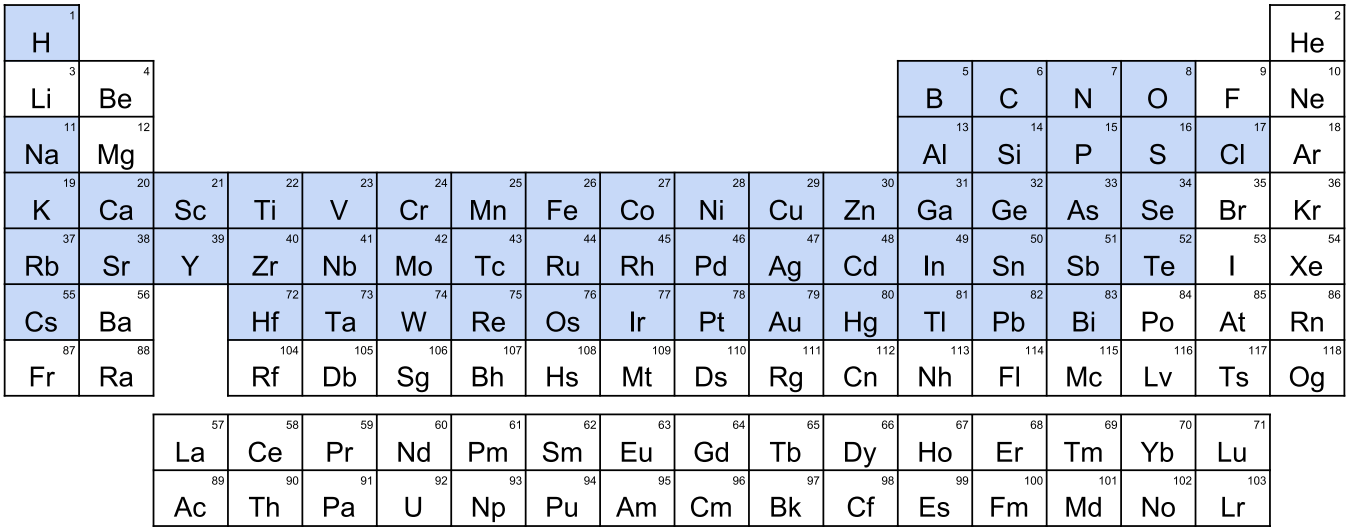

Supplementary Figure 6. The 56 elements supported by the OC20 dataset.

NOTE 9: NNP ARCHITECTURE AND INVARIANCES

In general, it is essential for machine learning models to incorporate the inductive bias of the target domain to improve the accuracy and generalization. Some of the properties imposed on NNs for atomic structures include rotational invariance, translational invariance, and mirror-image reversal invariance. Among them, the one with rotational invariance is called SO(N), that with translational invariance in addition to SO(N) is called SE(N), and the one with mirror-image inversion invariance in addition to SE(N) is called E(N). When they are not equipped, physically unnatural effects occur, such as unnatural external forces or the inferring of different energies for optical isomers.

However, from the viewpoint of improving the representational performance, there is a demand to design an architecture without losing higher-order features related to positional relationships. For example, in architectures based on atomic environment vectors (AEV) and node-based machine-learning potentials (MLPs) [2, 5–8], the positional relationship information is represented by the bond distances and angles. Rich local positional information is provided while preserving the invariance. However, because the message-passing mechanism of graph neural networks (GNNs) is lacking, positional information farther than the cutoff distance cannot be conveyed. Another problem is the explosion of a combination of elements. The angle term in the AEV has a number of combinations proportional to the cube of the type of element.

Various methods have been devised to handle higher-order structural information in GNN architectures. In Supplementary Table 4, we compare our architecture with previous studies in terms of invariance. These methods can be broadly classified into two categories: those that use spherical harmonics features and those that use vectors. The former has an invariance of SE(3), and the latter has an invariance of E(3). Among these, TeaNet [9] has E(3) invariance and can handle higher-order features such as second-order tensor quantities. Therefore, we adopted the TeaNet-style tensor-based convolution layer for our GNN architecture.

Architecture	Invariance	NN type	Scalar	sph	Vector	Tensor
BPNN [5]	$E(3)$ Invariant	MLP	✓			
ANI-1 [2]	$E(3)$ Invariant	MLP	✓			
ANI-2x [6]	$E(3)$ Invariant	MLP	✓			
Schrodinger-ANI [7]	$E(3)$ Invariant	MLP	✓			
TensorMol-0.1 [8]	$E(3)$ Invariant	MLP	✓			
SchNet [10]	$E(3)$ Invariant	GNN	✓			
DimeNet++ [11, 12]	$E(3)$ Invariant	GNN	✓			
PhysNet [13]	$E(3)$ Invariant	GNN	✓			
Cormorant [14]	$SE(3)$ Equivariant	GNN	✓	✓		
SE(3)-Transformer [15]	$SE(3)$ Equivariant	GNN	✓	✓		
NequIP [16]	$SE(3)$ Equivariant	GNN	✓	✓		
SpookyNet [17]	$SE(3)$ Equivariant	GNN	✓	✓		
EGNN [18]	$E(3)$ Equivariant	GNN	✓		✓	
TeaNet [9]	$E(3)$ Equivariant	GNN	✓		✓	✓
ours	$E(3)$ Equivariant	GNN	✓		✓	✓

Supplementary Table 4. Categorization of recent NNP architectures. Here, scalar denotes a rotation invariant feature, also often called an atomic environment vector (AEV) when used with MLPs. In addition, sph denotes a higher-order spherical harmonics feature, and vector and tensor represent first- and second-order rotational equivariant features, respectively. SE(3) models can also be turned into an E(3) model with additional constraints.

Note that when inputting a graph structure into an NN, nodes are transformed once into an ordered list, and NNPs generally need to also satisfy permutation invariance for the order of the nodes. All of the above models, including ours, satisfy this permutation invariance.

NOTE 10: STRUCTURE GENERATION DETAILS OF PFP DATASET

molecule

Base molecules were obtained from GDB-11 datasets [19, 20] or PubChem databases.[21] Additional base molecules were generated by modifying the elements of the base molecules. For example, diatomic molecules for all pairs of 45 elements were generated to obtain diatomic potentials. The structures of the molecules are generated by geometrical optimization, normal mode sampling (NMS) [2], or molecular dynamics. Structures with two molecules are also generated with molecular dynamics. The settings of NMS are approximately the same as those of the original method, but the number of structures to be sampled is changed to reduce the computational cost.

bulk

For the base structures of the bulk material, we gathered various single-element and binary-element crystal base structures with one or two elements for all 45 elements or pairs of 45 elements. For single-element crystal base structures, simple cubic, face-centered cubic, body-centered cubic, hexagonal close-packed, and diamond structures were prepared. One-dimensional chain structures and two-dimensional graphene structures were also prepared as single-element crystal base structures. For binary-element crystal base structures, zincblende, rocksalt, cesium chloride, and wurtzite structures were prepared. Furthermore, additional base structures were obtained from the materials project database [22] under the condition that the number of elements is two, the number of sites is less than or equal to 16, and at least one transition metal element (Ti − Zn, Mo, Ru − Ag) is included. The above base structures were volume-relaxed with DFT while maintaining the cell shapes and fractional coordinates of each site. Subsequently, additional deformed structures were generated for each base structure based on the volume-relaxed structure in four ways.

cell compression / expansion

We sampled 20 deformed structures for each base structure with this method. The cell volume was compressed or expanded while maintaining the cell shape and fractional coordinates of each site. In addition, 10 expanded structures were sampled for each base structure, where the relative cell length varied between 1.0 and 1.1. Another 10 structures were also sampled with lengths between 1.0 and 1.0/1.1.

site position displacement

For each base structure, 10 deformed structures were sampled with this method. The Cartesian coordinates of each site were randomly displaced while maintaining the cell shape and volume. The displacement distances of each site were obtained from the uniform distribution of 0 to 0.1 $\times$ (average distance between sites), and the displacement directions of each site were uniformly sampled.

cell shear deformation

Up to 60 deformed structures were sampled for each base structure with this method. Shear deformation was applied while maintaining the cell volume and the fractional coordinates of each site. A single parameter was selected from the lattice angle parameters α, β, and γ, while the other two were fixed. For each base structure, 21 structures were sampled by changing the selected angle parameter from -5° to $+5^\circ$ at even intervals. By removing the original structure, 20 structures were obtained. The corresponding shear plane was scaled to maintain the area. For example, in the case where the α-angle is selected, the b- and c-axis lengths are changed to maintain the area. Since there are three lattice angle parameters, $20 \times 3 = 60$ structures were generated from a one-volume relaxed structure. To reduce the calculation cost, symmetrically unique structures were selected from the 60 structures for the calculations using DFT.

cell tensile deformation

Up to 60 deformed structures were sampled for each base structure with this method. Tensile deformation was applied while maintaining the cell volume, cell angles, and fractional coordinates of each site. One of the a-, b-, or c-axis lengths was selected and scaled by arithmetic progression from 0.9 to 1.1, and 21 structures were obtained for each selected axis. By removing the original structure, 20 structures were obtained. The other unselected two axis lengths are also scaled to keep the cell volume. Symmetrically unique structures selected from 60 structures were calculated with DFT.

cluster

Small atomic clusters with one element were created based on 13-atom metal clusters.[23, 24] Covalent radii were used for the interatomic distance. The length of the vacuum region was 10 Å along each axis. To generate deformed structures, cell compression / expression method was applied to the cluster structures. The length of the vacuum region was kept constant during the deformation.

disordered

Structures that are far from stable configurations were sampled using molecular dynamics (MD) simulations at high temperatures. These structures are referred to as disordered structures. An interatomic potential is required to run MD simulations. To overcome this issue, we first created an early stage of PFP without the MD-origin structures and then used it to sample disordered structures. The calculation flow is as follows: First, the initial structures are created. A cubic cell is used for the initial structure. The typical cell length is 8.8 Å, but smaller cells with 8.4 Å and 8.0 Å are also prepared. The atoms are added in the form of a $2 \times 2 \times 2$ face-centered cubic structure (32 atoms) but with different element types. Multiple element selection strategies have been proposed. One method determines each atom type randomly. The maximum element type limit was set to 20. Another method selects 2, 4, or 8 element types repeatedly and feeds them into the cell. In this case, the number of atoms corresponding to each element was changed in units of 4 atoms. For example, the most unbalanced structure is 28 atoms in a certain element and 4 atoms in another element. The last method is similar to the previous one, but up to 24 atoms are eliminated from the structure. This means that the density of the structure is lower. Then, high-temperature molecular dynamics simulations were carried out. To unify the timescale of the atoms, the masses of all atoms were set to 5 (atomic mass unit). For the first step, the initial temperature was set to 6000 K, and then 1 ps of NPT ensemble at 10000 K was conducted. The second and third steps were 1 ps of the NPT ensemble at 2000 K and 500 K, respectively. Finally, to obtain the localized structure, the vacuum region was created by expanding one axis of the cell by a factor of two without modifying the atom positions, and 1 ps of NPT ensemble at 300 K was carried out. Four snapshots corresponding to the final state of the four processes were extracted and used for the disordered dataset structure. After DFT calculation, if the maximum atomic force is larger than 20 eV/Å, the structure is discarded.

slab

Slab structures with (111), (110), (101), (011), (100), (010), and (001) surfaces were generated from volume-relaxed bulk structures. The length of the vacuum region between the surfaces was 10 Å. The number of equivalent layers of the slab is typically 3. If the thickness of the 3-layer slab is too small or large, a larger or smaller number of layers was used, respectively. The site positions and cell parameters were not relaxed. Deformed structures were also generated from these slab structures. Four deformation methods introduced for bulk systems were applied to the slab structures. The number of generated deformed structures is halved when the computation cost is high. The length of the vacuum region was kept constant for the deformed structures.

adsorption

Adsorbed structures were generated for pairs of randomly selected slab structures and randomly selected molecules using the following two methods.

geometrical optimization

First, a randomly rotated molecule was placed at random positions in the vacuum region of the slab structure. Adoption sites, such as on-top, bridge, or hollow sites, were not considered when placing the molecule. Then, the adsorbed structure was geometrically optimized in the early stage of PFP while maintaining the cell parameters and site positions of all slab sites, including the sites on the surface. The definition of the early stage of PFP is described in the disordered section. Slab structures without deformation were selected for this method.

random placement

Initially, a randomly rotated molecule was placed at random positions in the vacuum region of the slab structure. Adoption sites, such as on-top, bridge, or hollow sites, were not considered when placing the molecule. Here, the closest atom is defined as the atom with the smallest distance normalized by its atomic radius. The normalized smallest distance d_{min} was calculated as follows: $d_{min} = \min_{i,j}(D_{ij}/(r_i + r_j))$, where i is the index of the slab atom; j is the index of the molecule atom; D_{ij} is the Cartesian distance between slab atom i and molecule atom j; r_i is the atomic radius of atom i; and, r_j is the atomic radius of atom j. The atomic radii used were determined by Slater[25] and are available on the Mendeleev package.[26] Then, the molecule is moved perpendicular to the slab surface so that the smallest normalized distance is the random value obtained from a uniform distribution in the range of 0.8 to 1.3. Slab structures without deformations or deformed slab structures with cell expansion/compression methods were selected for this method.

NOTE 11: RELATIONSHIP BETWEEN PFP APPLICATIONS AND DATASET

In this section, the correspondence between PFP applications shown in the results section and the dataset is demonstrated.

Lithium diffusion

The crystal structures of LiFeSO4F and FeSO4F are not explicitly included in the dataset. The crystal structures collected in the dataset (bulk section in Supplementary Note 10) are single-element and binary-element systems. This means that there are no three-body interactions of the three different elements in the crystal structure dataset. The nearest structure is likely included in the disordered dataset. It contains multiple types of elements, and there is a chance of having similar local configurations of FeSO4F or LiFeSO4F. The prerequisite knowledge of LiFeSO4F and FeSO4F was not used in the dataset collection phase.

Molecular adsorption in metal-organic framework

Although MOF structures shown in the results section are not explicitly included in the dataset, artificial molecule structures that contain non-organic elements are included in the dataset (molecule section in Supplementary Note 10). We believe that this is one of the structures closest to the dataset. Conversely, it only contains intramolecular interactions and does not have inter-molecular interactions, which have a major impact on the structure parameters of MOF. In addition, water molecule interactions were not explicitly included in the dataset. These intermolecular interactions may be observed in the adsorption dataset described in Supplementary Note 10.

Cu-Au alloy order-disorder transition

The ordered crystal lattices of the Cu-Au alloy are included in the dataset. Deformed and displaced structures are also included. However, the disordered crystal lattice observed in the simulation was not explicitly included in the dataset.

Material discovery for a Fischer–Tropsch catalyst

The potential curve of the CO molecule is also included in the dataset. The adsorption dataset and external OC20 dataset contain adsorbed structures. However, they correspond to the adsorption energy, instead of the bond-breaking process of the molecules. The catalyst effect of the surfaces shown in the results section was not explicitly included in the dataset.

NOTE 12: PFP APPLICATIONS COMPARISON WITH OC20 DIMENET++ MODEL

To check the performance of the PFP shown in the results section, the results are compared with those of the existing NNP. The Open Catalyst Project baseline model is used for this task because it was trained on a recently proposed large-scale dataset and applies to multi-element systems.

The publicly available trained model dimenetpp_all is available from the Open Catalyst Project implementation (https://github.com/Open-Catalyst-Project/ocp/tree/v0.0.3). This model was trained with the DimeNet++[12] architecture on all data from the S2EF task[1] included in OC20.

In this study, two modifications were made to the dimenetpp_all inference implementation. First, the cell input was modified to ensure differentiability to compute the stress tensor with automatic differentiation to subsequently use it for cell structure optimization. Second, the upper limit of the number of neighbors considered in the GNN was increased from 50 to 150 to avoid losing the continuity of the energy landscape. We noticed that the estimated energy fluctuated during the structural optimization process when the maximum number of neighbors was 50, which is the default setting used for the training process. It was confirmed that increasing the maximum number of neighbors up to 150 suppresses this energy fluctuation, and the structural optimization converges successfully.

The functional use for the DFT calculations is different. For PFP, the dataset is collected based on the PBE functional and compared with previous studies in the main text. Conversely, the OC20 dataset is calculated using the RPBE functional.[27] A direct comparison of the results requires careful consideration.

Lithium diffusion

It is not possible to optimize the structure of $FeSO_4F$ using dimenetpp_all because of energy fluctuations. Therefore, we compared the energy for the same NEB images obtained using PFP. The energy in the initial structure was set to zero in both models. The results are presented in Fig. 7; dimenetpp_all sometimes fails to reproduce the existence of an energy barrier. Notably, the lithium diffusion phenomenon in the bulk structure is outside the scope of OC20 tasks.

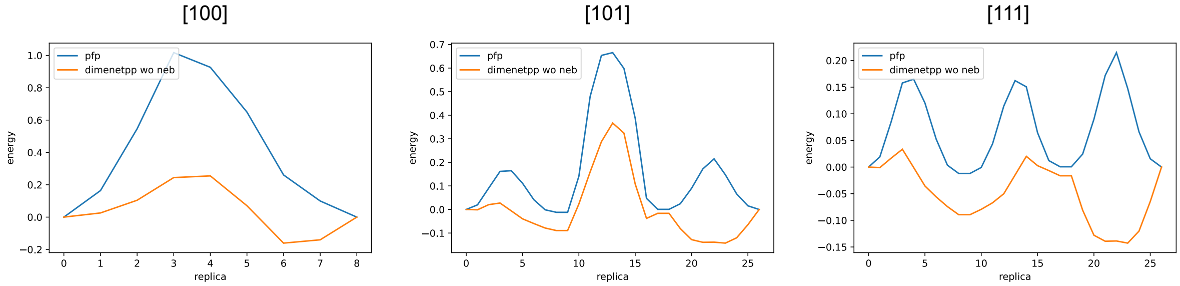

Supplementary Figure 7. NEB calculation images v.s. energies by PFP (blue line). All images were evaluated using dimenetpp_all (orange line).

metal-organic framework

Since the structural optimization does not converge, neither the volume change rate nor the adsorption energy could be evaluated by dimenetpp_all.

Cu-Au alloy

It was determined that the bulk energy evaluated by dimenetpp_all is not appropriate for reproducing the transition phenomena. For example, the formation energy of the CuAu rocksalt structure was quite small (10^{-7} eV/atom order), which is not consistent with the DFT calculation (0.1 eV/atom order, using the PBE functional).

Fischer–Tropsch catalyst

NEB calculations were performed using dimenetpp_all. The same optimization method and NEB conditions were applied. Although the energy values of PFP and dimenetpp_all differ, the diagrams are relatively reasonable and can be considered an interpolation region of the OC20 dataset.

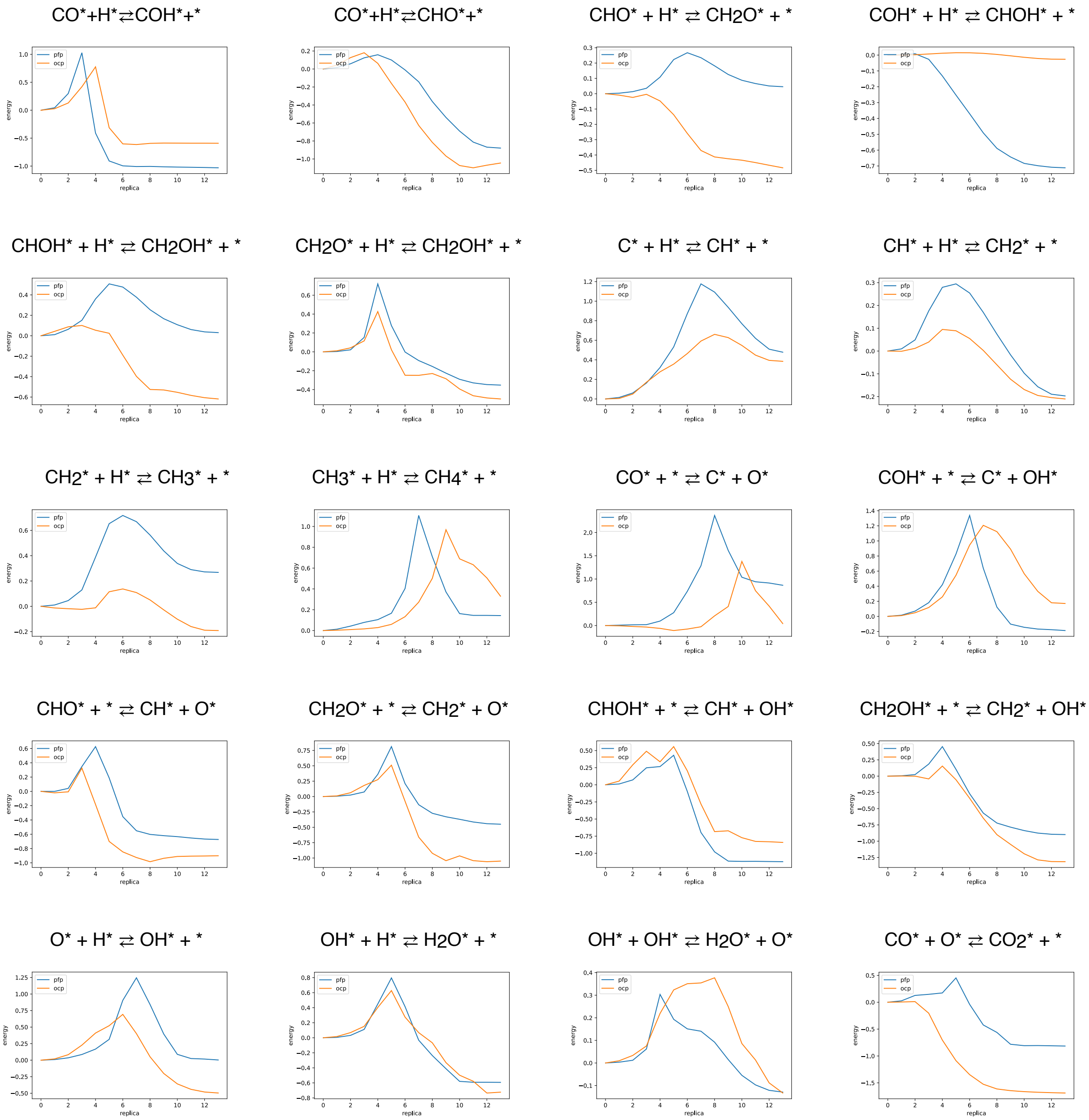

Supplementary Figure 8. Comparison of the NEB calculation result. The blue and orange lines correspond to PFP and dimenetpp_all, respectively. The energy in the initial state was set to zero.

It was difficult to reproduce the adsorption process using dimenetpp_all. To create an adsorbed structure, the molecule was attached to the optimized surface (Fig. 9 (a)). However, the structure obtained is not reasonable (Fig. 9 (b)). Although the adsorbed structure can be obtained by attaching the molecule to the bare surface, where both of them can be optimized simultaneously (Fig. 9 (c)), the evaluated energy indicates that structure b (-6.15 eV) is more stable than structure c (-1.56 eV).

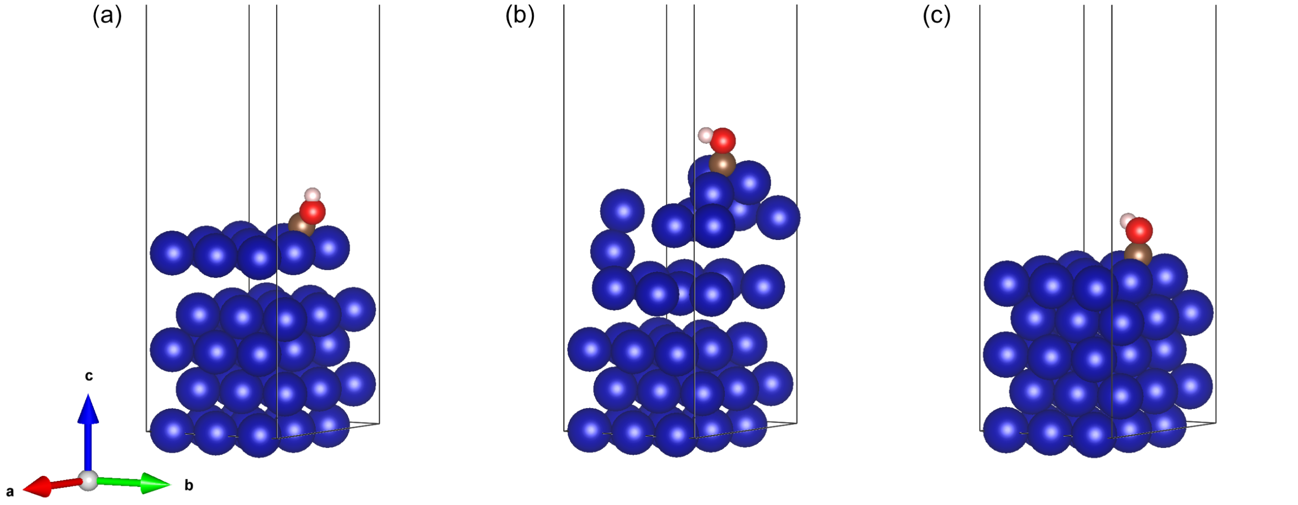

Supplementary Figure 9. Adsorbed structures created by dimenetpp_all. (a) COH molecule attached to the optimized Co surface (b) Optimized structure of (a). (c) Structure optimized from the COH molecule placed on the Co surface. The figures were drawn using the VESTA visualization package.[28]

NOTE 13: MOLECULAR DYNAMICS SIMULATION OF LITHIUM DIFFUSION

The detail of the calculation method for the activation energy of lithium diffusion using molecular dynamics simulation is shown below.

First, the same structure as the initial state of the NEB calculation was prepared. Second, the initial momenta were applied based on the Maxwell-Boltzmann distribution. Then, 100 ps of the NVT ensemble was applied at a constant temperature. The temperature was set at 300 K, 325 K, 350 K, 375 K, and 400 K. Eight independent trajectories were sampled for each temperature by changing the random seed of the initial momenta. It was verified that the lithium atom only travels along the [111] direction, which is considered the lowest activation energy path for the CI-NEB calculations.

The trajectory of the lithium atom was sampled every 0.01 ps for 80 ps, starting 20 ps after the initial state of the MD simulation. The mean squared distance (MSD) of the lithium atom along the [111] direction was calculated by changing the time span from 0.05 ps to 10 ps, and the diffusion coefficient was calculated by fitting the linear coefficient of the MSD with respect to the time span.[29] The Arrhenius plot is shown in Fig. 10. The fitted line was calculated from the series of the mean diffusion coefficients at each temperature. The activation energy corresponded to the coefficient of the fitted line, which was determined to be 0.202 eV for the activation energy of lithium diffusion.

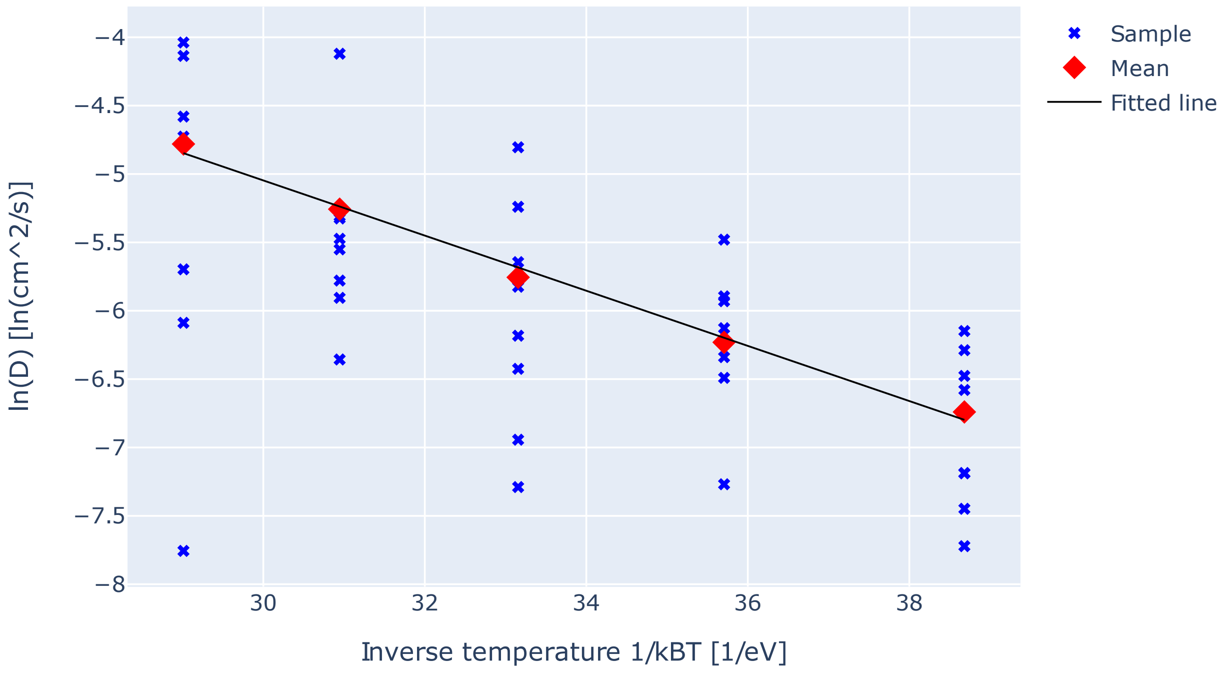

Supplementary Figure 10. Arrhenius plot of lithium diffusion coefficient. One trajectory at 325 K has a very small diffusion coefficient (8.2×10^{-7} cm^2/s), which is not plotted in this figure.

NOTE 14: COMPUTATIONAL DETAILS OF MOF

The crystalline structures of some representative MOFs were optimized using PFP. The starting crystalline structures were obtained from the Cambridge Structure Database (CSD).[30] The initial structures were cleaned by removing the physically adsorbed molecules in the pores of the MOFs. Water molecules that are chemically bound to the metal centers were maintained. We call these structures hydrated structures. Other minor cleansing procedures were performed by adding hydrogen atoms and removing overlapping atoms to ensure physically reasonable crystal structures and stoichiometries. Geometry optimization was performed on each MOF to determine the lowest energy configuration. The convergence criterion for the geometry optimization is for the maximum force on any atom to reach below 5 meV/Å. The Broyden – Fletcher – Goldfarb – Shanno (BFGS) algorithm was used to optimize both the cell geometry and atom positions.[31] Supplementary Figure 11 shows the crystal structure of hydrated MOF-74-Mg.

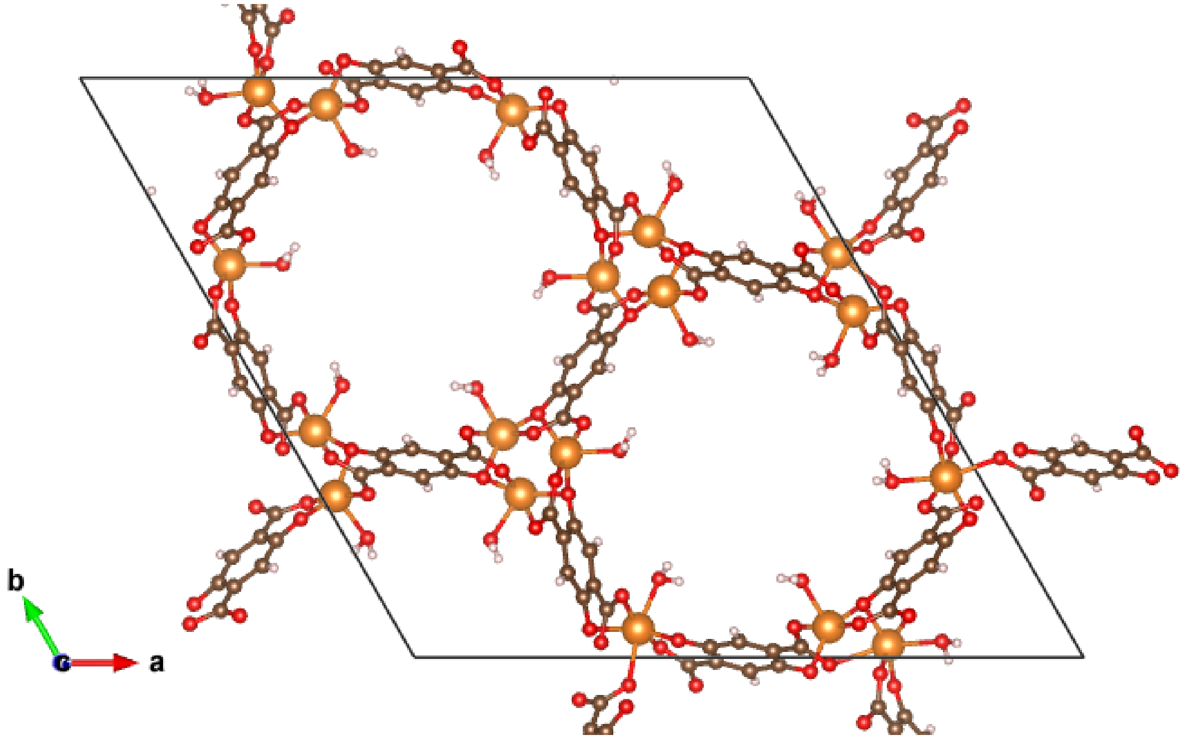

Supplementary Figure 11. Crystal structure of hydrated MOF-74-Mg viewed along the c-axis. Elements are represented by red (oxygen), orange (magnesium), gray (carbon), and white (hydrogen).

NOTE 15: COMPARISON OF INDIVIDUAL CELL PARAMETERS OF MOFS CALCULATED BY PFP

Supplementary Table 5 shows the comparison of individual cell parameters of MOFs calculated by PFP.

Supplementary Table 5. Unit cell parameters and volumes of the selected MOFs. Each experimental crystal structure was identified using the Cambridge Structure Database (CSD) identifier.[30] ΔV_{exp} represents the relative volumetric error of the PFP and PFP+D3 optimized geometries relative to the experimentally reported crystal structures.

Metal	CSD identifier	Data type	a (Å)	b (Å)	c (Å)	α (deg.)	β (deg.)	γ (deg.)	V (Å^3)	ΔV_{exp} (%)
MOF-5	SAHYIK	PFP	26.20	26.20	26.20	90.0	90.0	90.0	17982	6.32
		PFP+D3	26.15	26.15	26.15	90.0	90.0	90.0	17874	5.68
		Exp.[32]	25.67	25.67	25.67	90.0	90.0	90.0	16913	
Cu-BTC	FIQCEN	PFP	26.50	26.47	26.47	90.0	89.9	90.1	18566	1.56
		PFP+D3	26.42	26.34	26.35	90.0	89.8	90.1	18337	0.30
		Exp.[33]	26.34	26.34	26.34	90.0	90.0	90.0	18281	
ZIF-90	WOJGEI	PFP	17.28	17.28	17.28	90.0	90.0	90.0	5161	0.17
		PFP+D3	17.13	17.13	17.13	90.0	90.0	90.0	5026	-2.45
		Exp.[34]	17.27	17.27	17.27	90.0	90.0	90.0	5152	
MIL-53-Al	SAVUN	PFP	6.73	17.27	13.07	90.0	90.0	90.0	1517	7.47
		PFP+D3	6.70	17.21	13.04	90.0	90.0	90.0	1505	6.62
		Exp.[35]	6.61	16.68	12.81	90.0	90.0	90.0	1412	
MOF-74-Mg	VOGTIV	PFP	26.20	26.21	7.05	90.0	90.0	120.0	4194	5.79
		PFP+D3	26.10	26.10	7.01	90.0	90.0	120.0	4132	4.23
		Exp.[36]	26.03	26.03	6.76	90.0	90.0	120.0	3965	
MOF-74-Co	NAVJAW	PFP	26.13	26.13	7.04	90.0	90.0	120.0	4164	4.97
		PFP+D3	26.01	26.01	6.94	90.0	90.0	120.0	4064	2.46
		Exp.[37]	26.11	26.11	6.72	90.0	90.0	120.0	3967	
MOF-74-Ni	LEJRIC	PFP	26.05	26.04	6.97	90.0	90.0	120.0	4093	4.71
		PFP+D3	25.89	25.88	6.88	90.0	90.0	120.0	3993	2.16
		Exp.[38]	25.98	25.98	6.69	90.0	90.0	120.0	3909	
MOF-74-Zn	WOBHEB	PFP	26.24	26.24	7.00	90.0	90.0	120.0	4176	4.87
		PFP+D3	26.11	26.11	6.95	90.0	90.0	120.0	4103	3.03
		Exp.[39]	26.26	26.26	6.67	90.0	90.0	120.0	3983	

NOTE 16: METROPOLIS SAMPLING METHOD

The MC moves used in the Cu-Au alloy order-disorder transition simulation were conducted using Metropolis sampling.[40], such that an arbitrary pair of atoms is swapped and the structure is relaxed, and the energy change (ΔE) is recorded. Since the optimization of the structure is computationally expensive, it is performed only at every 100 steps. Then, the Boltzmann factor, $\exp\left(-\Delta E/k_B T\right)$, is computed and compared with a randomly selected number between 0 and 1 according to a uniform distribution used to determine whether the move should be accepted or rejected. If the random number is smaller than the Boltzmann factor, the move is accepted; otherwise, the move is rejected. The MC loop was iterated over 200,000 steps to ensure equilibrium. The final structure of the MC runs was characterized based on the Voronoi-weighted Steinhardt parameters.[41] These parameters are suitable for characterizing the ordering of the atomic arrangement. To calculate under periodic conditions, a 10 Å vacuum was applied along each axis. To reduce the computational cost, a 7 Å vacuum was also used.

NOTE 17: HIGH-TEMPERATURE MULTI-ELEMENT DATASET DESCRIPTION

In this study, we provide an atomic structure dataset called the high-temperature multi-element 2021 (HME21) dataset, which consists of a portion of the PFP dataset.[42]

There are several reasons for providing HME21. The first reason is enabling readers to further analyze the properties of PFP by providing a portion of the PFP dataset. We believe that the nature of the dataset, such as the number of element types in a single structure, or the variety of neighboring atoms, plays an important role in achieving the molecular dynamics simulation results shown in this study. The second reason is to be a standard benchmark for future universal NNP development. As mentioned in the Introduction, existing datasets were generated based on known structures, such as the molecule or crystal structures. During our exploration while building the new dataset, we noticed that the existing NNP architectures did not behave as expected when the dataset components were far from stable atomic structures. In most cases, the hyperparameters must be returned to proceed with the training process. We believe that the HME21 dataset will serve as a guide for the design of future universal NNPs. The third reason is to provide an NNP architecture benchmark combined with the dataset, allowing readers to confirm the benchmark results themselves. The corresponding benchmark results of the NNP-architecture are presented in Supplementary Note 18.

HME21 corresponds to the disordered dataset described in Supplementary Note 10. We regarded this subcomponent as a typical structure in the PFP dataset. It contains multiple elements in a single structure and was sampled through a high-temperature molecular dynamics simulation. Thus, the structures are far from stable and contain less prior specific domain knowledge, such as the molecule or crystal structures. The structures were sampled from the entire disordered dataset and randomly split into training, validation, and test sub-datasets at a ratio of 8:1:1. The numbers of structures in these datasets are 19956, 2498, and 2495, respectively. There are a total of 37 elements in the HME21 dataset, i.e., H, Li, C, N, O, F, Na, Mg, Al, Si, P, S, Cl, K, Ca, Sc, Ti, V, Cr, Mn, Fe, Co, Ni, Cu, Zn, Mo, Ru, Rh, Pd, Ag, In, Sn, Ba, Ir, Pt, Au, and Pb. Each dataset contains structural information (element types, atomic positions, and cell shapes) and target values (energy and atomic forces). All structures are under periodic boundary conditions. The energy is shifted such that the energy of a single atom located in a vacuum becomes zero. The length is in ångströms (10^{-10} m), and the energy is in electronvolts (eV).

The details of the DFT calculation conditions are provided in Supplementary Note 10.

NOTE 18: NEURAL NETWORK ARCHITECTURE BENCHMARK USING HME21

To show the performances of NNP architectures for multi-element structures that are far from having a stable state, we applied a benchmark using the HME21 dataset (see Supplementary Note 17). For this benchmark, we selected TeaNet[9], SchNet [10], PaiNN [43], and NequIP [16]. TeaNet corresponds to the base model of PFP. It treats tensor representations as higher-order geometric features. SchNet uses the bond length for spatial information and employs a convolution with rotationally invariant filters. This has been well examined using various datasets, and its limited representation power has been discussed. PaiNN incorporates a vector representation to resolve the problem of a limited representation of rotationally invariant filters of SchNet. On the other hand, NequIP uses spherical harmonics-based representations. The experimental code for both SchNet and PaiNN is based on the repository found at https://github.com/learningmatter-mit/NeuralForceField, whereas the experimental code for NequiP is based on the repository found at https://github.com/mir-group/nequip.

Next, we discuss the choice of hyperparameter. To optimize the performance with respect to the validation set, the hyperparameter selection procedure is based on a grid search and manual hyperparameter tuning. For TeaNet, we use a four-layer model. We first set the energy loss coefficient c_{le} (energy per atom MSE) to 0.0001 and retrained it using $c_{le} = 1.0$ and $c_{le} = 10.0$, whereas the force loss coefficient c_{lf} remained constant at 1.0. The batch size was set to 16, and the learning rate was initialized to 0.001. For SchNet, we use a four-layer model, where the energy loss coefficient was set to 0.05, the batch size was set to 32, and the learning rate was initialized to 0.0005. For PaiNN, we use a three-layer model, where the energy loss coefficient was set to 0.05, the batch size was set to 32, and the learning rate was initialized to 0.0005. For NequIP, we use a five-layer model with different maximum rotation orders $l_{\max} \in \{0, 1, 2\}$. For the five-layer model, the energy loss coefficient was set to 0.01 and the learning rate was initialized to 0.001. For $l_{\max} \in \{0, 1\}$, we found that setting the batch size to 32 worked best, whereas for $l_{\max} = 2$, setting the batch size to 64 was preferable. We set the cutoff distance to 6.0 Åfor all architectures.

The results are presented in Supplementary Table 6. TeaNet performed well in terms of both the energy and force metrics, which indicates that the TeaNet architecture is suitable for multielement structures which are far from stable coordination.

The results, including the implementation of the TeaNet architecture, are available along with those of the HME21 dataset (Supplementary Data 2).

Architecture	Energy MAE [meV/atom]	Force MAE [eV/Å]	Force XYZ MAE [eV/Å]
TeaNet (PFP base model)	19.6	0.174	0.153
SchNet	33.6	0.283	0.247
PaiNN	22.9	0.237	0.208
NequIP ($l_{\max} = 0$)	52.2	0.249	0.225
NequIP ($l_{\max} = 1$)	53.3	0.233	0.206
NequIP ($l_{\max} = 2$)	47.8	0.199	0.175

Supplementary Table 6. Benchmark performance of NNP for the force and energy prediction for the HME21 dataset. Energy MAE corresponds to the mean absolute error of the energies of structures divided by their numbers of atoms, Force MAE corresponds to the mean absolute error of the norm of force vectors, and Force XYZ MAE corresponds to the mean absolute error of the force vector component.

NOTE 19: STATISTICAL INFORMATION OF PFP DATASET

Supplementary Table 7 lists the statistical information of the PFP molecule and PFP crystal datasets.

Supplementary Table 7. Statistical information of (a) PFP molecule and (b) PFP crystal datasets. Here, # of atoms and # of elements correspond to the numbers of atoms and elements in each structure, respectively. In addition, Energy, corresponding to the energy of the structures divided by their number of atoms, and the energy of single atoms in a vacuum, is set to zero. Force corresponds to the L^2 norm of the force vector of each atom. The range, mean, and standard deviation (SD) are shown.

(a) PFP molecule dataset

	# of	# of atoms			# of elements			Energy [eV/atom]		Force [eV/Å]	
Structure	Structure	Range	Mean	SD	Range	Mean	SD	Mean	SD	Mean	SD
Optimize	2.6×10^5	2 – 26	15	3.4	1 – 9	4.1	1.0	-4.1	0.35	1.4×10^{-5}	5.8×10^{-3}
NMS	4.6×10^6	2 – 26	15	3.6	1 – 9	3.8	0.86	-4.0	0.35	3.8	6.7
MD	7.8×10^5	2 – 26	16	3.6	1 – 6	3.7	0.74	-4.0	0.32	7.7	15
MD with two molecules	4.4×10^5	4 – 40	23	5.2	1 – 6	4.5	0.80	-3.9	0.31	3.8	12

(b) PFP Crystal Dataset

	# of	# of atoms			# of elements			Energy [eV/atom]		Force [eV/Å] ,	
Structure	Structure	Range	Mean	SD	Range	Mean	SD	Mean	SD	Mean	SD
Molecule	1.0×10^6	2 – 36	13	5.3	1 – 9	4.0	1.2	-2.8	1.2	2.6	7.1
Bulk, cell compression / expansion	5.9×10^4	2 – 16	5.5	4.2	1 – 2	1.9	0.28	-3.1	1.3	0.91	5.5
Bulk, site position displacement	3.2×10^4	4 – 112	15	7.7	1 – 2	1.9	0.25	-2.9	1.3	15	39
Bulk, cell shear deformation	4.4×10^4	2 – 16	5.3	4.0	1 – 2	2.0	0.19	-3.2	1.3	0.18	0.60
Bulk, cell tensile deformation	6.0×10^4	2 – 16	5.7	4.2	1 – 2	2.0	0.16	-3.2	1.3	0.61	3.4
Cluster	2.2×10^4	13 – 13	13	0.0	1 – 1	1.0	0.0	-1.7	1.2	11	33
Disordered	8.1×10^5	2 – 128	27	19	1 – 28	7.8	6.2	-2.5	0.99	11	26
Slab	7.4×10^5	3 – 192	18	10	1 – 2	1.9	0.24	-2.9	1.3	1.3	5.8
Adsorption, geometrical optimization	9.7×10^4	6 – 121	33	19	2 – 8	4.3	0.86	-3.5	0.91	0.76	8.2
Adsorption, random placement	2.8×10^5	5 – 400	52	25	1 – 6	4.5	0.78	-3.1	0.93	2.9	10

NOTE 20: PFP MOLECULE MODE WITH OUT-OF-DOMAIN ELEMENTS

In this section, the domain transferability of the PFP under different DFT conditions is examined. We compared the molecule structures of phenol, lithium phenoxide, and sodium phenoxide estimated using both PFP with molecule mode and PFP with crystal mode. In crystal mode, all elements are included in the dataset, and it is expected that all three of these molecules are in-domain molecules. Supplementary Figure 12 shows the optimized structures obtained using crystal mode. By contrast, in molecule mode, C, H, and O are included in the dataset but Li and Na are not. Therefore, if an NNP is trained using a molecule dataset only, it is clear that there is no information at all regarding Li and Na elements, and the structural estimations of lithium phenoxide and sodium phenoxide will fail. However, PFP was concurrently trained using both the molecule and crystal datasets. This means that, even under molecule mode inference, PFP knows the behaviors of Li and Na atoms in crystal mode, and there is a possibility of transferring them to molecule mode inference.

Comparisons of the bond and angle parameters of the optimized structures of these three molecules between the crystal and molecule modes are shown in Supplementary Table 8. First, the positions of the Li and Na atoms were consistent in both the crystal and molecule modes. It can be said that the behaviors of Li and Na atoms under molecule mode imitate those of crystal mode. Second, the ratios of the bond distances in the benzene ring (C–C, C–H, C–O) between the crystal and molecule modes were mostly constant among the three molecules. Owing to the difference in DFT conditions, the bond distances of phenol are slightly different between the molecule and crystal modes. It can be interpreted that the benzene rings of lithium phenoxide and sodium phenoxide follow the molecule dataset.

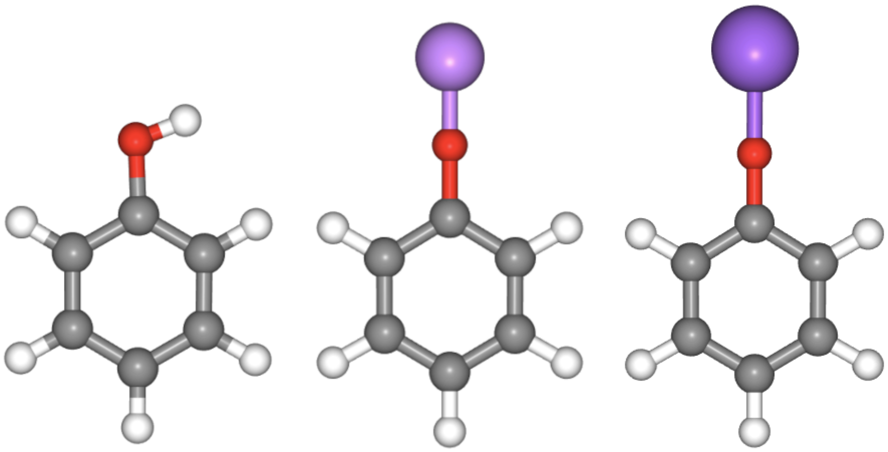

Supplementary Figure 12. Optimized structures of phenol, lithium phenoxide, and sodium phenoxide using PFP with crystal mode.

	Crystal mode			Molecule mode			Ratio		
	H	Li	Na	H	Li	Na	H	Li	Na
C–C bond min [Å]	1.396	1.396	1.396	1.390	1.391	1.390	1.004	1.004	1.005
C–C bond max [Å]	1.402	1.416	1.423	1.398	1.413	1.419	1.003	1.002	1.003
C–H bond min [Å]	1.091	1.092	1.092	1.086	1.087	1.087	1.005	1.005	1.005
C–H bond max [Å]	1.094	1.093	1.094	1.089	1.087	1.088	1.005	1.006	1.006
C–O bond [Å]	1.379	1.336	1.323	1.362	1.313	1.303	1.012	1.017	1.015
O–X bond [Å]	0.973	1.618	1.980	0.963	1.618	2.016	1.010	1.000	0.982
C–O–X angle [degree]	109.395	179.996	179.645	109.574	179.997	179.610	0.998	1.000	1.000

Supplementary Table 8. Bond distances and angles of optimized structures of phenol, lithium phenoxide, and sodium phenoxide estimated using PFP with crystal mode and PFP with molecule mode. The H, Li, and Na columns correspond to phenol, lithium phenoxide, and sodium phenoxide, respectively. In addition, bond min and bond max correspond to the minimum and maximum bond distances of the corresponding bonds, respectively; and X corresponds to the H, Li, and Na atoms in the hydroxy group. Finally, the ratio column shows the ratio of values between crystal and molecule modes.

SUPPLEMENTARY REFERENCES

[1] Lowik Chanussot, Abhishek Das, Siddharth Goyal, Thibaut Lavril, Muhammed Shuaibi, Morgane Riviere, Kevin Tran, Javier Heras-Domingo, Caleb Ho, Weihua Hu, Aini Palizhati, Anuroop Sriram, Brandon Wood, Junwoong Yoon, Devi Parikh, C. Lawrence Zitnick, and Zachary Ulissi. Open catalyst 2020 (oc20) dataset and community challenges. ACS Catalysis, 11(10):6059–6072, 2021. doi:10.1021/acscatal.0c04525. URL `https://doi.org/10.1021/acscatal.0c04525`.

[2] J. S. Smith, O. Isayev, and A. E. Roitberg. Ani-1: an extensible neural network potential with dft accuracy at force field computational cost. Chem. Sci., 8:3192–3203, 2017. doi:10.1039/C6SC05720A. URL `http://dx.doi.org/10.1039/C6SC05720A`.

[3] Paolo Giannozzi, Stefano Baroni, Nicola Bonini, Matteo Calandra, Roberto Car, Carlo Cavazzoni, Davide Ceresoli, Guido L Chiarotti, Matteo Cococcioni, Ismaila Dabo, Andrea Dal Corso, Stefano de Gironcoli, Stefano Fabris, Guido Fratesi, Ralph Gebauer, Uwe Gerstmann, Christos Gougoussis, Anton Kokalj, Michele Lazzeri, Layla Martin-Samos, Nicola Marzari, Francesco Mauri, Riccardo Mazzarello, Stefano Paolini, Alfredo Pasquarello, Lorenzo Paulatto, Carlo Sbraccia, Sandro Scandolo, Gabriele Sclauzero, Ari P Seitsonen, Alexander Smogunov, Paolo Umari, and Renata M Wentzcovitch. Quantum espresso: a modular and open-source software project for quantum simulations of materials. Journal of Physics: Condensed Matter, 21(39):395502 (19pp), 2009. URL `http://www.quantum-espresso.org`.

[4] P Giannozzi, O Andreussi, T Brumme, O Bunau, M Buongiorno Nardelli, M Calandra, R Car, C Cavazzoni, D Ceresoli, M Cococcioni, N Colonna, I Carnimeo, A Dal Corso, S de Gironcoli, P Delugas, R A DiStasio Jr, A Ferretti, A Floris, G Fratesi, G Fugallo, R Gebauer, U Gerstmann, F Giustino, T Gorni, J Jia, M Kawamura, H-Y Ko, A Kokalj, E Küçükbenli, M Lazzeri, M Marsili, N Marzari, F Mauri, N L Nguyen, H-V Nguyen, A Otero de-la Roza, L Paulatto, S Poncé, D Rocca, R Sabatini, B Santra, M Schlipf, A P Seitsonen, A Smogunov, I Timrov, T Thonhauser, P Umari, N Vast, X Wu, and S Baroni. Advanced capabilities for materials modelling with quantum espresso. Journal of Physics: Condensed Matter, 29(46):465901, 2017. URL `http://stacks.iop.org/0953-8984/29/i=46/a=465901`.

[5] Jörg Behler. Constructing high-dimensional neural network potentials: A tutorial review. International Journal of Quantum Chemistry, 115(16):1032–1050, 2015. doi:https://doi.org/10.1002/qua.24890. URL `https://onlinelibrary.wiley.com/doi/abs/10.1002/qua.24890`.

[6] Christian Devereux, Justin S. Smith, Kate K. Davis, Kipton Barros, Roman Zubatyuk, Olexandr Isayev, and Adrian E. Roitberg. Extending the applicability of the ani deep learning molecular potential to sulfur and halogens. Journal of Chemical Theory and Computation, 16(7):4192–4202, 2020. doi:10.1021/acs.jctc.0c00121. URL `https://doi.org/10.1021/acs.jctc.0c00121`. PMID: 32543858.

[7] James M. Stevenson, Leif D. Jacobson, Yutong Zhao, Chuanjie Wu, Jon Maple, Karl Leswing, Edward Harder, and Robert Abel. Schrödinger-ani: An eight-element neural network interaction potential with greatly expanded coverage of druglike chemical space, 2019.

[8] Kun Yao, John E. Herr, David W. Toth, Ryker Mckintyre, and John Parkhill. The tensormol-0.1 model chemistry: a neural network augmented with long-range physics. Chem. Sci., 9:2261–2269, 2018. doi:10.1039/C7SC04934J. URL `http://dx.doi.org/10.1039/C7SC04934J`.

[9] So Takamoto, Satoshi Izumi, and Ju Li. Teanet: Universal neural network interatomic potential inspired by iterative electronic relaxations. Computational Materials Science, 207:111280, 2022. ISSN 0927-0256. doi:https://doi.org/10.1016/j.commatsci.2022.111280. URL `https://www.sciencedirect.com/science/article/pii/S0927025622000799`.

[10] K. T. Schütt, P.-J. Kindermans, H. E. Sauceda, S. Chmiela, A. Tkatchenko, and K.-R. Müller. Schnet: A continuous-filter convolutional neural network for modeling quantum interactions. In Proceedings of the 31st International Conference on Neural Information Processing Systems, Red Hook, NY, USA, 2017. Curran Associates Inc. ISBN 9781510860964.

[11] Johannes Klicpera, Janek Groß, and Stephan Günnemann. Directional message passing for molecular graphs. In International Conference on Learning Representations, 2020. URL `https://openreview.net/forum?id=B1eWbxStPH`.

[12] Johannes Klicpera, Shankari Giri, Johannes T. Margraf, and Stephan Günnemann. Fast and uncertainty-aware directional message passing for non-equilibrium molecules. In NeurIPS-W, 2020.

[13] Oliver T. Unke and Markus Meuwly. Physnet: A neural network for predicting energies, forces, dipole moments, and partial charges. Journal of Chemical Theory and Computation, 15(6):3678–3693, 2019. doi:10.1021/acs.jctc.9b00181. URL `https://doi.org/10.1021/acs.jctc.9b00181`. PMID: 31042390.

[14] Brandon Anderson, Truong Son Hy, and Risi Kondor. Cormorant: Covariant molecular neural networks. In H. Wallach, H. Larochelle, A. Beygelzimer, F. d'Alché-Buc, E. Fox, and R. Garnett, editors, Advances in Neural Information Processing Systems, volume 32. Curran Associates, Inc., 2019. URL `https://proceedings.neurips.cc/paper/2019/file/03573b32b2746e6e8ca98b9123f2249b-Paper.pdf`.

[15] Fabian Fuchs, Daniel Worrall, Volker Fischer, and Max Welling. Se(3)-transformers: 3d roto-translation equivariant attention networks. In H. Larochelle, M. Ranzato, R. Hadsell, M. F. Balcan, and H. Lin, editors, Advances in Neural Information Processing Systems, volume 33, pages 1970–1981. Curran Associates, Inc., 2020. URL `https://proceedings.neurips.cc/paper/2020/file/15231a7ce4ba789d13b722cc5c955834-Paper.pdf`.

[16] Simon Batzner, Tess E. Smidt, Lixin Sun, Jonathan P. Mailoa, Mordechai Kornbluth, Nicola Molinari, and Boris Kozinsky.

Se(3)-equivariant graph neural networks for data-efficient and accurate interatomic potentials, 2021.

[17] Oliver T. Unke, Stefan Chmiela, Michael Gastegger, Kristof T. Schütt, Huziel E. Sauceda, and Klaus-Robert Müller. Spookynet: Learning force fields with electronic degrees of freedom and nonlocal effects, 2021.

[18] Victor Garcia Satorras, Emiel Hoogeboom, and Max Welling. E(n) equivariant graph neural networks, 2021.

[19] Tobias Fink and Jean-Louis Reymond. Virtual exploration of the chemical universe up to 11 atoms of c, n, o, f: assembly of 26.4 million structures (110.9 million stereoisomers) and analysis for new ring systems, stereochemistry, physicochemical properties, compound classes, and drug discovery. Journal of chemical information and modeling, 47(2):342–353, 2007.

[20] Tobias Fink, Heinz Bruggesser, and Jean-Louis Reymond. Virtual exploration of the small-molecule chemical universe below 160 daltons. Angewandte Chemie International Edition, 44(10):1504–1508, 2005.

[21] Eric W Sayers, Jeffrey Beck, Evan E Bolton, Devon Bourexis, James R Brister, Kathi Canese, Donald C Comeau, Kathryn Funk, Sunghwan Kim, William Klimke, Aron Marchler-Bauer, Melissa Landrum, Stacy Lathrop, Zhiyong Lu, Thomas L Madden, Nuala O' Leary, Lon Phan, Sanjida H Rangwala, Valerie A Schneider, Yuri Skripchenko, Jiyao Wang, Jian Ye, Barton W Trawick, Kim D Pruitt, and Stephen T Sherry. Database resources of the National Center for Biotechnology Information. Nucleic Acids Research, 49(D1):D10–D17, 10 2020. ISSN 0305-1048. doi:10.1093/nar/gkaa892. URL https://doi.org/10.1093/nar/gkaa892.

[22] Anubhav Jain, Shyue Ping Ong, Geoffroy Hautier, Wei Chen, William Davidson Richards, Stephen Dacek, Shreyas Cholia, Dan Gunter, David Skinner, Gerbrand Ceder, and Kristin a. Persson. The Materials Project: A materials genome approach to accelerating materials innovation. APL Materials, 1(1):011002, 2013. ISSN 2166532X. doi:10.1063/1.4812323. URL http://link.aip.org/link/AMPADS/v1/i1/p011002/s1\&Agg=doi.

[23] Yan Sun, René Fournier, and Min Zhang. Structural and electronic properties of 13-atom $4d$ transition-metal clusters. Phys. Rev. A, 79:043202, Apr 2009. doi:10.1103/PhysRevA.79.043202. URL https://link.aps.org/doi/10.1103/PhysRevA.79.043202.

[24] Min Zhang and René Fournier. Density-functional-theory study of 13-atom metal clusters M_{13}, $m =$ Ta−−Pt. Phys. Rev. A, 79:043203, Apr 2009. doi:10.1103/PhysRevA.79.043203. URL https://link.aps.org/doi/10.1103/PhysRevA.79.043203.

[25] John C Slater. Atomic radii in crystals. The Journal of Chemical Physics, 41(10):3199–3204, 1964.

[26] Łukasz Mentel. mendeleev – a python resource for properties of chemical elements, ions and isotopes. URL https://github.com/lmmentel/mendeleev.

[27] B Hammer, L B Hansen, and J K Nørskov. Improved adsorption energetics within density-functional theory using revised Perdew-Burke-Ernzerhof functionals. Phys. Rev. B Condens. Matter, 59(11):7413–7421, March 1999.

[28] Koichi Momma and Fujio Izumi. Vesta 3 for three-dimensional visualization of crystal, volumetric and morphology data. Journal of applied crystallography, 44(6):1272–1276, 2011.

[29] Gaurav Pranami and Monica H. Lamm. Estimating error in diffusion coefficients derived from molecular dynamics simulations. Journal of Chemical Theory and Computation, 11(10):4586–4592, 2015. doi:10.1021/acs.jctc.5b00574. URL https://doi.org/10.1021/acs.jctc.5b00574.

[30] Colin R. Groom, Ian J. Bruno, Matthew P. Lightfoot, and Suzanna C. Ward. The Cambridge Structural Database. Acta Crystallographica Section B, 72(2):171–179, Apr 2016. doi:10.1107/S2052520616003954. URL https://doi.org/10.1107/S2052520616003954.

[31] Hiroyasu Furukawa, Kyle E. Cordova, Michael O'Keeffe, and Omar M. Yaghi. The chemistry and applications of metal-organic frameworks. Science, 341(6149), 2013. ISSN 0036-8075. doi:10.1126/science.1230444. URL https://science.sciencemag.org/content/341/6149/1230444.

[32] Mohamed Eddaoudi, Jaheon Kim, Nathaniel Rosi, David Vodak, Joseph Wachter, Michael O'Keeffe, and Omar M. Yaghi. Systematic design of pore size and functionality in isoreticular mofs and their application in methane storage. Science, 295 (5554):469–472, 2002. doi:10.1126/science.1067208.

[33] Stephen S.-Y. Chui, Samuel M.-F. Lo, Jonathan P. H. Charmant, A. Guy Orpen, and Ian D. Williams. A chemically functionalizable nanoporous material [cu3 (tma) 2 (h2o) 3] n. Science, 283(5405):1148–1150, 1999. doi:10.1126/science.283.5405.1148.

[34] William Morris, Christian J. Doonan, Hiroyasu Furukawa, Rahul Banerjee, and Omar M. Yaghi. Crystals as molecules: Postsynthesis covalent functionalization of zeolitic imidazolate frameworks. Journal of the American Chemical Society, 130(38):12626–12627, 2008. doi:10.1021/ja805222x. URL https://doi.org/10.1021/ja805222x. PMID: 18754585.

[35] Thierry Loiseau, Christian Serre, Clarisse Huguenard, Gerhard Fink, Francis Taulelle, Marc Henry, Thierry Bataille, and Gérard Férey. A rationale for the large breathing of the porous aluminum terephthalate (mil-53) upon hydration. Chemistry – A European Journal, 10(6):1373–1382, 2004. doi:https://doi.org/10.1002/chem.200305413. URL https://chemistry-europe.onlinelibrary.wiley.com/doi/abs/10.1002/chem.200305413.

[36] Pascal D. C. Dietzel, Richard Blom, and Helmer Fjellvåg. Base-induced formation of two magnesium metal-organic framework compounds with a bifunctional tetratopic ligand. European Journal of Inorganic Chemistry, 2008(23):3624–3632, 2008. doi:https://doi.org/10.1002/ejic.200701284. URL https://chemistry-europe.onlinelibrary.wiley.com/doi/abs/10.1002/ejic.200701284.

[37] Pascal D. C. Dietzel, Yusuke Morita, Richard Blom, and Helmer Fjellvåg. An in situ high-temperature single-crystal investigation of a dehydrated metal – organic framework compound and field-induced magnetization of one-dimensional metal – oxygen chains. Angewandte Chemie International Edition, 44(39):6354–6358, 2005. doi:https://doi.org/10.1002/anie.200501508. URL https://onlinelibrary.wiley.com/doi/abs/10.1002/anie.200501508.

[38] Pascal D. C. Dietzel, Barbara Panella, Michael Hirscher, Richard Blom, and Helmer Fjellvåg. Hydrogen adsorption in a nickel based coordination polymer with open metal sites in the cylindrical cavities of the desolvated framework. Chem. Commun., pages 959–961, 2006. doi:10.1039/B515434K. URL http://dx.doi.org/10.1039/B515434K.

[39] Pascal D. C. Dietzel, Rune E. Johnsen, Richard Blom, and Helmer Fjellvåg. Structural changes and coordinatively unsaturated metal atoms on dehydration of honeycomb analogous microporous metal – organic frameworks. Chemistry – A European Journal, 14(8):2389–2397, 2008. doi:https://doi.org/10.1002/chem.200701370. URL https://chemistry-europe.onlinelibrary.wiley.com/doi/abs/10.1002/chem.200701370.

[40] Daan Frenkel and Berend Smit. Understanding Molecular Simulation From Algorithms to Applications. Academic Press, 2 edition, 2001. ISBN 9780080519982.

[41] Walter Mickel, Sebastian C. Kapfer, Gerd E. Schröder-Turk, and Klaus Mecke. Shortcomings of the bond orientational order parameters for the analysis of disordered particulate matter. The Journal of Chemical Physics, 138(4):044501, 2013. doi:10.1063/1.4774084. URL https://doi.org/10.1063/1.4774084.

[42] So Takamoto, Chikashi Shinagawa, Daisuke Motoki, Kosuke Nakago, Wenwen Li, Iori Kurata, Taku Watanabe, Yoshihiro Yayama, Hiroki Iriguchi, Yusuke Asano, Tasuku Onodera, Takafumi Ishii, Takao Kudo, Hideki Ono, Ryohto Sawada, Ryuichiro Ishitani, Marc Ong, Taiki Yamaguchi, Toshiki Kataoka, Akihide Hayashi, Nontawat Charoenphakdee, and Takeshi Ibuka. High-temperature multi-element 2021 (HME21) dataset. 4 2022. doi:10.6084/m9.figshare.19658538. URL https://figshare.com/articles/dataset/High-temperature_multi-element_2021_HME21_dataset/19658538.

[43] Kristof Schütt, Oliver Unke, and Michael Gastegger. Equivariant message passing for the prediction of tensorial properties and molecular spectra. In International Conference on Machine Learning, pages 9377–9388. PMLR, 2021.
