# Supplementary material for: Towards universal neural network potential for material discovery applicable to arbitrary combination of 45 elements
Source: Nat Commun. 2022 May 30;13:2991. doi: 10.1038/s41467-022-30687-9 (PMC9151783; doi:10.1038/s41467-022-30687-9)
Supplement: Supplementary file 3 — Description of Additional Supplementary Files [file 41467_2022_30687_MOESM3_ESM.docx]

Description of Additional Supplementary Files

File name: Supplementary Data 1

Description: Simulation script files and output data corresponding to the result section.

File name: Supplementary Data 2

Description: Code for NNP architecture benchmark using HME21 including TeaNet implementation with the trained parameters.
