# Supplementary figures and images for: Towards universal neural network potential for material discovery applicable to arbitrary combination of 45 elements
Source: Nat Commun. 2022 May 30;13:2991. doi: 10.1038/s41467-022-30687-9 (PMC9151783; doi:10.1038/s41467-022-30687-9)

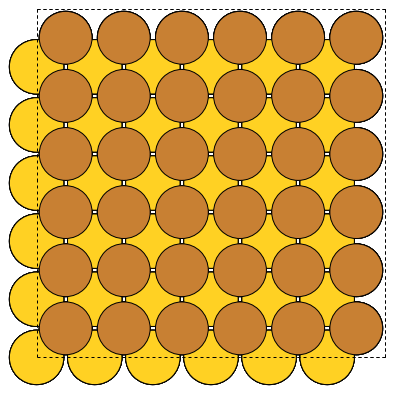

Supplement: Supplementary file 4 — Supplementary Data 1 [file 41467_2022_30687_MOESM4_ESM.zip › scripts/3_od_transition/OD-transition/CuAu/CuAu_4x4x4_00600K.png]

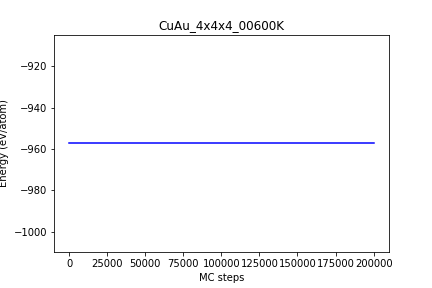

Supplement: Supplementary file 4 — Supplementary Data 1 [file 41467_2022_30687_MOESM4_ESM.zip › scripts/3_od_transition/OD-transition/CuAu/CuAu_4x4x4_00600K_energy.png]

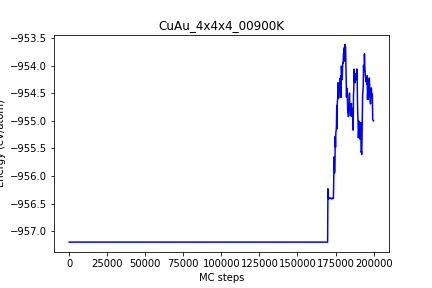

Supplement: Supplementary file 4 — Supplementary Data 1 [file 41467_2022_30687_MOESM4_ESM.zip › scripts/3_od_transition/OD-transition/CuAu/CuAu_4x4x4_00900K_energy.png]

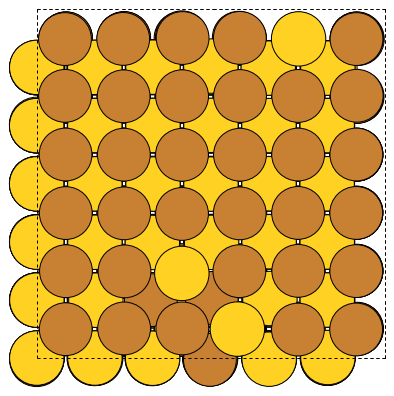

Supplement: Supplementary file 4 — Supplementary Data 1 [file 41467_2022_30687_MOESM4_ESM.zip › scripts/3_od_transition/OD-transition/CuAu/CuAu_4x4x4_00900K.png]

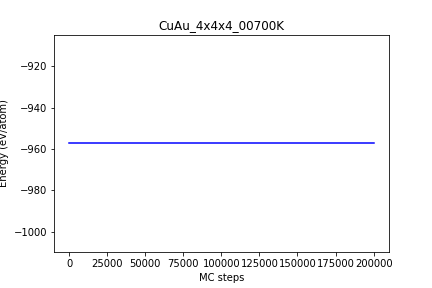

Supplement: Supplementary file 4 — Supplementary Data 1 [file 41467_2022_30687_MOESM4_ESM.zip › scripts/3_od_transition/OD-transition/CuAu/CuAu_4x4x4_00700K_energy.png]

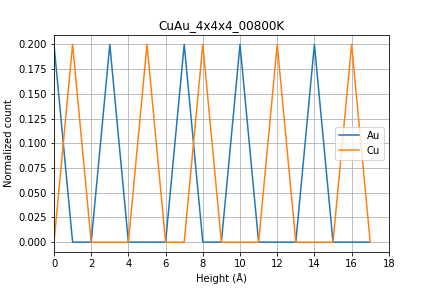

Supplement: Supplementary file 4 — Supplementary Data 1 [file 41467_2022_30687_MOESM4_ESM.zip › scripts/3_od_transition/OD-transition/CuAu/CuAu_4x4x4_00800K_depth_profile.png]

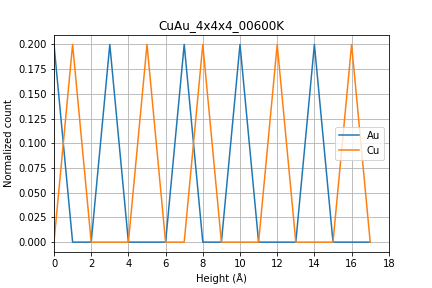

Supplement: Supplementary file 4 — Supplementary Data 1 [file 41467_2022_30687_MOESM4_ESM.zip › scripts/3_od_transition/OD-transition/CuAu/CuAu_4x4x4_00600K_depth_profile.png]

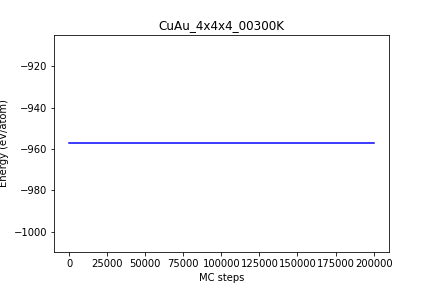

Supplement: Supplementary file 4 — Supplementary Data 1 [file 41467_2022_30687_MOESM4_ESM.zip › scripts/3_od_transition/OD-transition/CuAu/CuAu_4x4x4_00300K_energy.png]

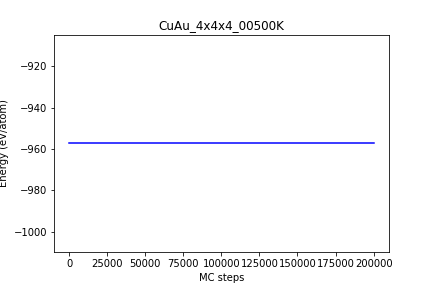

Supplement: Supplementary file 4 — Supplementary Data 1 [file 41467_2022_30687_MOESM4_ESM.zip › scripts/3_od_transition/OD-transition/CuAu/CuAu_4x4x4_00500K_energy.png]

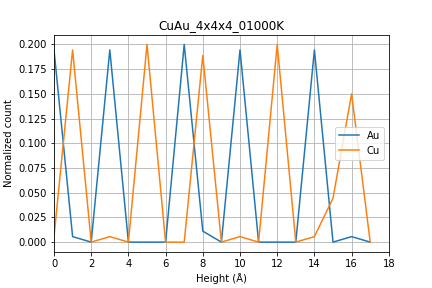

Supplement: Supplementary file 4 — Supplementary Data 1 [file 41467_2022_30687_MOESM4_ESM.zip › scripts/3_od_transition/OD-transition/CuAu/CuAu_4x4x4_01000K_depth_profile.png]

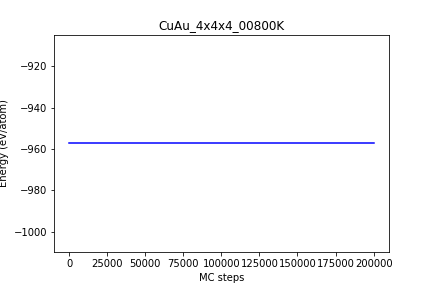

Supplement: Supplementary file 4 — Supplementary Data 1 [file 41467_2022_30687_MOESM4_ESM.zip › scripts/3_od_transition/OD-transition/CuAu/CuAu_4x4x4_00800K_energy.png]

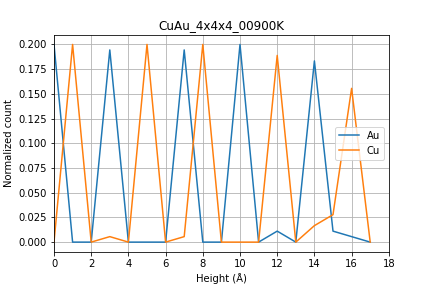

Supplement: Supplementary file 4 — Supplementary Data 1 [file 41467_2022_30687_MOESM4_ESM.zip › scripts/3_od_transition/OD-transition/CuAu/CuAu_4x4x4_00900K_depth_profile.png]

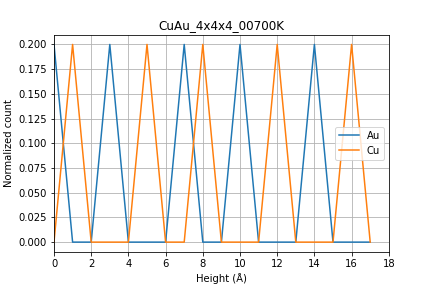

Supplement: Supplementary file 4 — Supplementary Data 1 [file 41467_2022_30687_MOESM4_ESM.zip › scripts/3_od_transition/OD-transition/CuAu/CuAu_4x4x4_00700K_depth_profile.png]

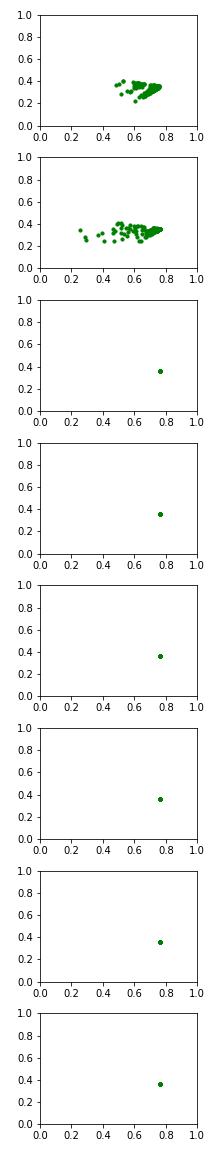

Supplement: Supplementary file 4 — Supplementary Data 1 [file 41467_2022_30687_MOESM4_ESM.zip › scripts/3_od_transition/OD-transition/CuAu/CuAu_Cu_q4q6.png]

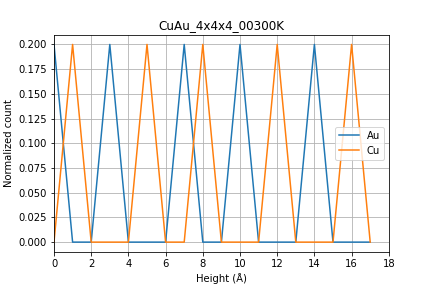

Supplement: Supplementary file 4 — Supplementary Data 1 [file 41467_2022_30687_MOESM4_ESM.zip › scripts/3_od_transition/OD-transition/CuAu/CuAu_4x4x4_00300K_depth_profile.png]

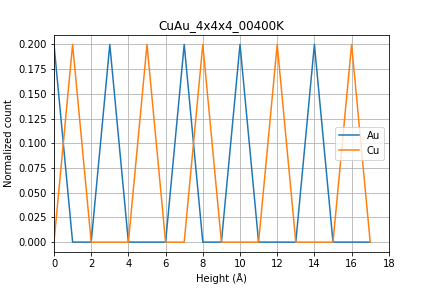

Supplement: Supplementary file 4 — Supplementary Data 1 [file 41467_2022_30687_MOESM4_ESM.zip › scripts/3_od_transition/OD-transition/CuAu/CuAu_4x4x4_00400K_depth_profile.png]

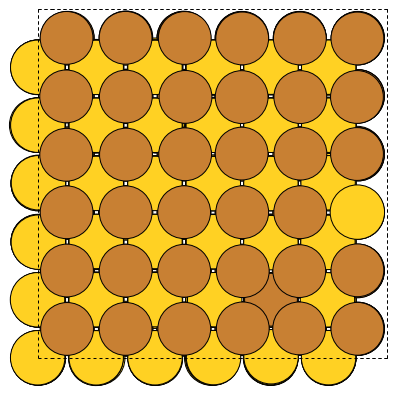

Supplement: Supplementary file 4 — Supplementary Data 1 [file 41467_2022_30687_MOESM4_ESM.zip › scripts/3_od_transition/OD-transition/CuAu/CuAu_4x4x4_01000K.png]

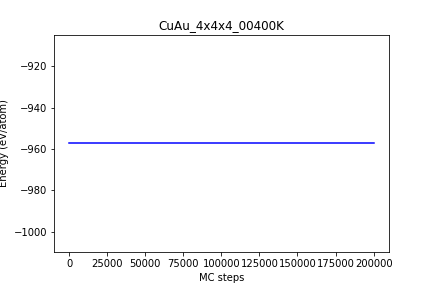

Supplement: Supplementary file 4 — Supplementary Data 1 [file 41467_2022_30687_MOESM4_ESM.zip › scripts/3_od_transition/OD-transition/CuAu/CuAu_4x4x4_00400K_energy.png]

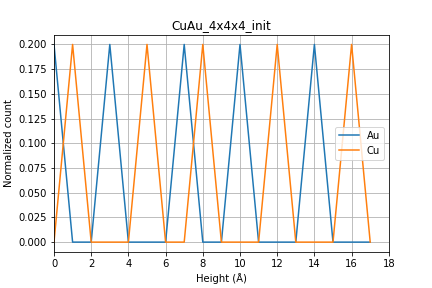

Supplement: Supplementary file 4 — Supplementary Data 1 [file 41467_2022_30687_MOESM4_ESM.zip › scripts/3_od_transition/OD-transition/CuAu/CuAu_4x4x4_init_depth_profile.png]

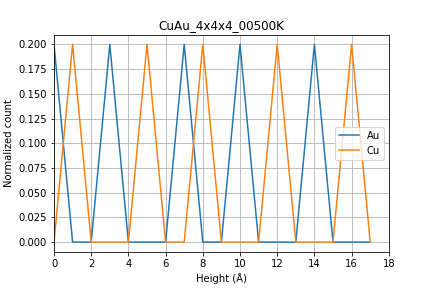

Supplement: Supplementary file 4 — Supplementary Data 1 [file 41467_2022_30687_MOESM4_ESM.zip › scripts/3_od_transition/OD-transition/CuAu/CuAu_4x4x4_00500K_depth_profile.png]

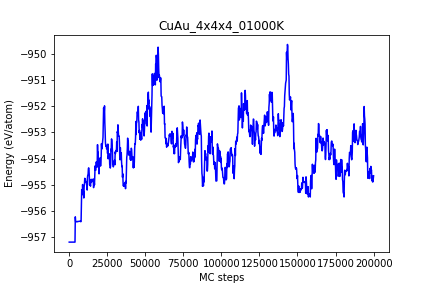

Supplement: Supplementary file 4 — Supplementary Data 1 [file 41467_2022_30687_MOESM4_ESM.zip › scripts/3_od_transition/OD-transition/CuAu/CuAu_4x4x4_01000K_energy.png]

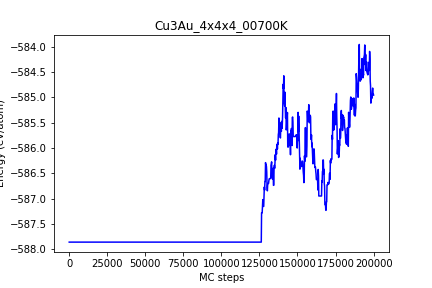

Supplement: Supplementary file 4 — Supplementary Data 1 [file 41467_2022_30687_MOESM4_ESM.zip › scripts/3_od_transition/OD-transition/Cu3Au/Cu3Au_4x4x4_00700K_energy.png]

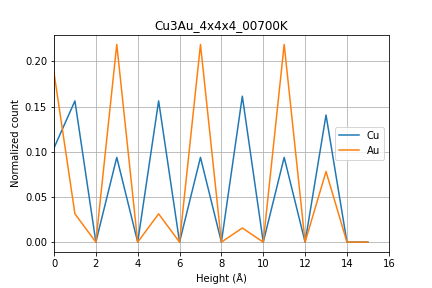

Supplement: Supplementary file 4 — Supplementary Data 1 [file 41467_2022_30687_MOESM4_ESM.zip › scripts/3_od_transition/OD-transition/Cu3Au/Cu3Au_4x4x4_00700K_depth_profile.png]

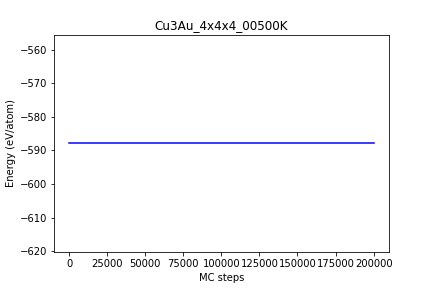

Supplement: Supplementary file 4 — Supplementary Data 1 [file 41467_2022_30687_MOESM4_ESM.zip › scripts/3_od_transition/OD-transition/Cu3Au/Cu3Au_4x4x4_00500K_energy.png]

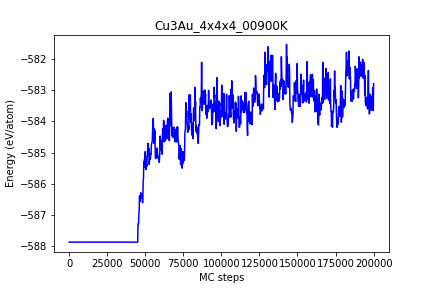

Supplement: Supplementary file 4 — Supplementary Data 1 [file 41467_2022_30687_MOESM4_ESM.zip › scripts/3_od_transition/OD-transition/Cu3Au/Cu3Au_4x4x4_00900K_energy.png]

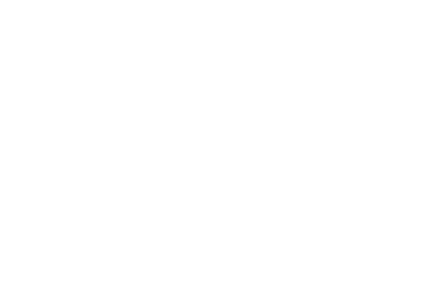

Supplement: Supplementary file 4 — Supplementary Data 1 [file 41467_2022_30687_MOESM4_ESM.zip › scripts/3_od_transition/OD-transition/Cu3Au/Cu3Au_Au_q4q6.png]

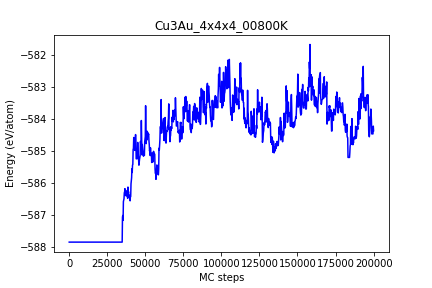

Supplement: Supplementary file 4 — Supplementary Data 1 [file 41467_2022_30687_MOESM4_ESM.zip › scripts/3_od_transition/OD-transition/Cu3Au/Cu3Au_4x4x4_00800K_energy.png]

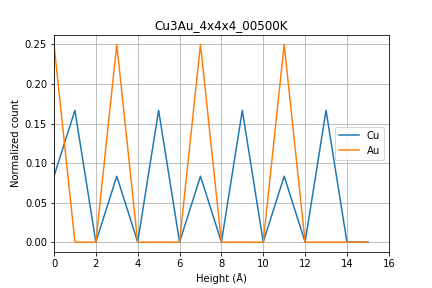

Supplement: Supplementary file 4 — Supplementary Data 1 [file 41467_2022_30687_MOESM4_ESM.zip › scripts/3_od_transition/OD-transition/Cu3Au/Cu3Au_4x4x4_00500K_depth_profile.png]

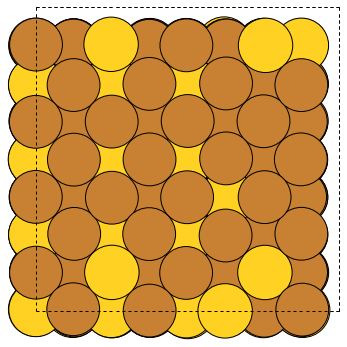

Supplement: Supplementary file 4 — Supplementary Data 1 [file 41467_2022_30687_MOESM4_ESM.zip › scripts/3_od_transition/OD-transition/Cu3Au/Cu3Au_4x4x4_00700K.png]

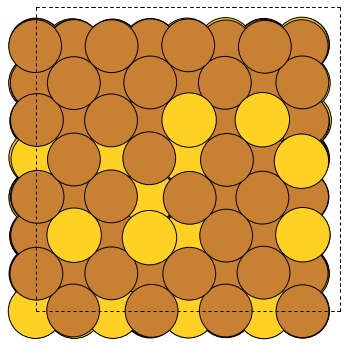

Supplement: Supplementary file 4 — Supplementary Data 1 [file 41467_2022_30687_MOESM4_ESM.zip › scripts/3_od_transition/OD-transition/Cu3Au/Cu3Au_4x4x4_00900K.png]

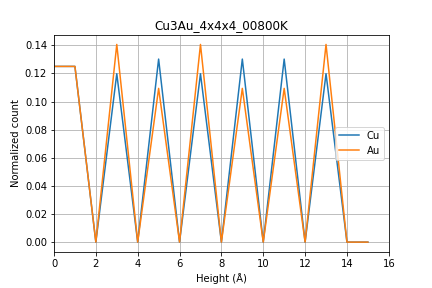

Supplement: Supplementary file 4 — Supplementary Data 1 [file 41467_2022_30687_MOESM4_ESM.zip › scripts/3_od_transition/OD-transition/Cu3Au/Cu3Au_4x4x4_00800K_depth_profile.png]

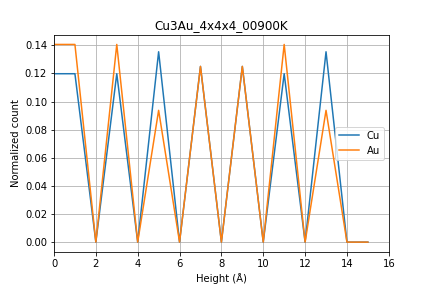

Supplement: Supplementary file 4 — Supplementary Data 1 [file 41467_2022_30687_MOESM4_ESM.zip › scripts/3_od_transition/OD-transition/Cu3Au/Cu3Au_4x4x4_00900K_depth_profile.png]

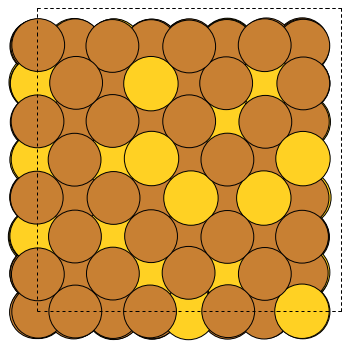

Supplement: Supplementary file 4 — Supplementary Data 1 [file 41467_2022_30687_MOESM4_ESM.zip › scripts/3_od_transition/OD-transition/Cu3Au/Cu3Au_4x4x4_01000K.png]

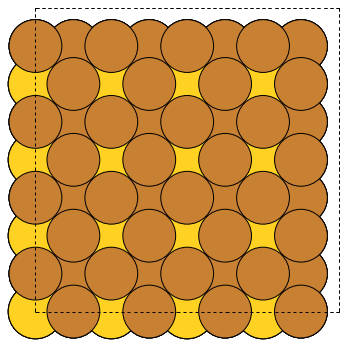

Supplement: Supplementary file 4 — Supplementary Data 1 [file 41467_2022_30687_MOESM4_ESM.zip › scripts/3_od_transition/OD-transition/Cu3Au/Cu3Au_4x4x4_00300K.png]

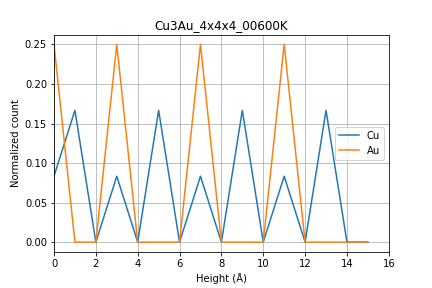

Supplement: Supplementary file 4 — Supplementary Data 1 [file 41467_2022_30687_MOESM4_ESM.zip › scripts/3_od_transition/OD-transition/Cu3Au/Cu3Au_4x4x4_00600K_depth_profile.png]

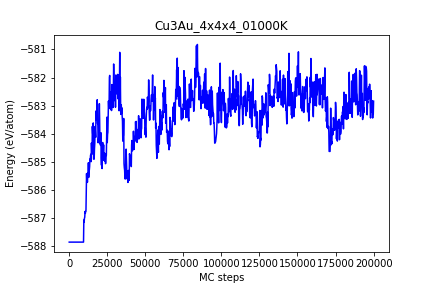

Supplement: Supplementary file 4 — Supplementary Data 1 [file 41467_2022_30687_MOESM4_ESM.zip › scripts/3_od_transition/OD-transition/Cu3Au/Cu3Au_4x4x4_01000K_energy.png]

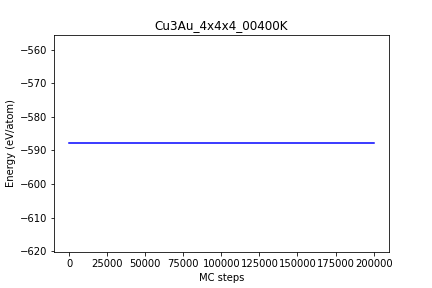

Supplement: Supplementary file 4 — Supplementary Data 1 [file 41467_2022_30687_MOESM4_ESM.zip › scripts/3_od_transition/OD-transition/Cu3Au/Cu3Au_4x4x4_00400K_energy.png]

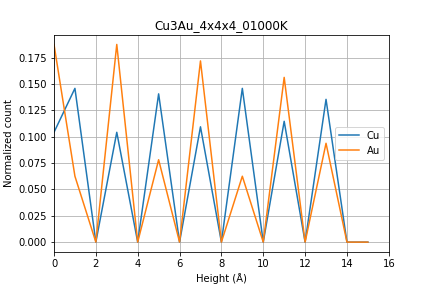

Supplement: Supplementary file 4 — Supplementary Data 1 [file 41467_2022_30687_MOESM4_ESM.zip › scripts/3_od_transition/OD-transition/Cu3Au/Cu3Au_4x4x4_01000K_depth_profile.png]

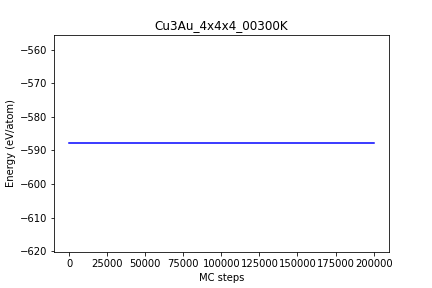

Supplement: Supplementary file 4 — Supplementary Data 1 [file 41467_2022_30687_MOESM4_ESM.zip › scripts/3_od_transition/OD-transition/Cu3Au/Cu3Au_4x4x4_00300K_energy.png]

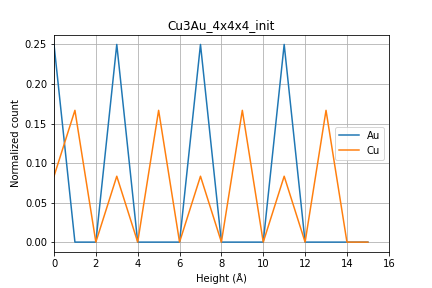

Supplement: Supplementary file 4 — Supplementary Data 1 [file 41467_2022_30687_MOESM4_ESM.zip › scripts/3_od_transition/OD-transition/Cu3Au/Cu3Au_4x4x4_init_depth_profile.png]

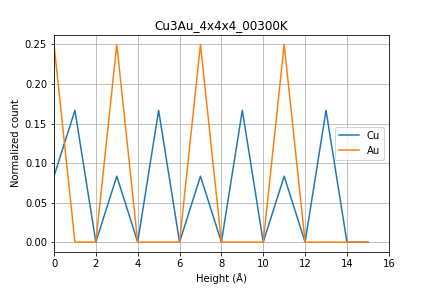

Supplement: Supplementary file 4 — Supplementary Data 1 [file 41467_2022_30687_MOESM4_ESM.zip › scripts/3_od_transition/OD-transition/Cu3Au/Cu3Au_4x4x4_00300K_depth_profile.png]

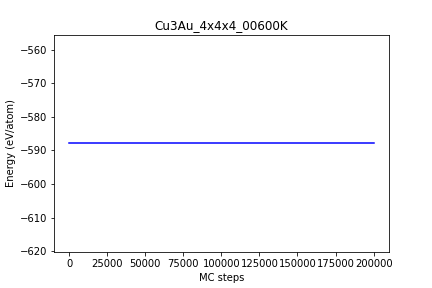

Supplement: Supplementary file 4 — Supplementary Data 1 [file 41467_2022_30687_MOESM4_ESM.zip › scripts/3_od_transition/OD-transition/Cu3Au/Cu3Au_4x4x4_00600K_energy.png]

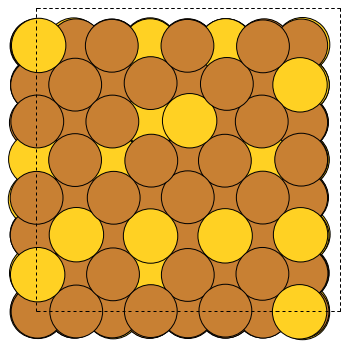

Supplement: Supplementary file 4 — Supplementary Data 1 [file 41467_2022_30687_MOESM4_ESM.zip › scripts/3_od_transition/OD-transition/Cu3Au/Cu3Au_4x4x4_00800K.png]

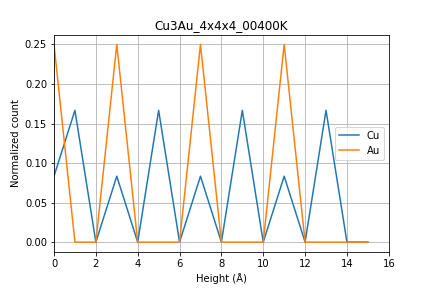

Supplement: Supplementary file 4 — Supplementary Data 1 [file 41467_2022_30687_MOESM4_ESM.zip › scripts/3_od_transition/OD-transition/Cu3Au/Cu3Au_4x4x4_00400K_depth_profile.png]

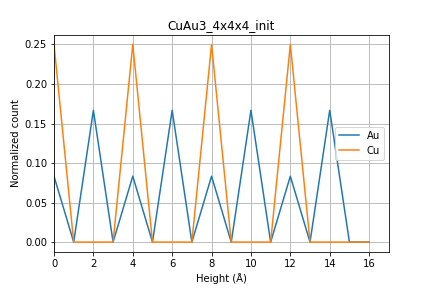

Supplement: Supplementary file 4 — Supplementary Data 1 [file 41467_2022_30687_MOESM4_ESM.zip › scripts/3_od_transition/OD-transition/CuAu3/CuAu3_4x4x4_init_depth_profile.png]

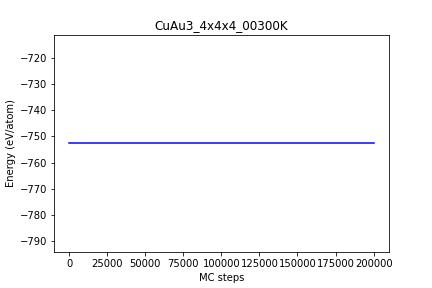

Supplement: Supplementary file 4 — Supplementary Data 1 [file 41467_2022_30687_MOESM4_ESM.zip › scripts/3_od_transition/OD-transition/CuAu3/CuAu3_4x4x4_00300K_energy.png]

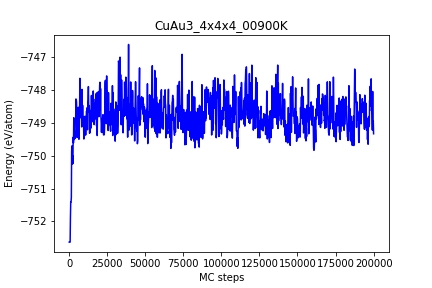

Supplement: Supplementary file 4 — Supplementary Data 1 [file 41467_2022_30687_MOESM4_ESM.zip › scripts/3_od_transition/OD-transition/CuAu3/CuAu3_4x4x4_00900K_energy.png]

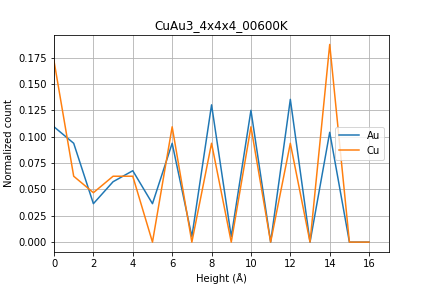

Supplement: Supplementary file 4 — Supplementary Data 1 [file 41467_2022_30687_MOESM4_ESM.zip › scripts/3_od_transition/OD-transition/CuAu3/CuAu3_4x4x4_00600K_depth_profile.png]

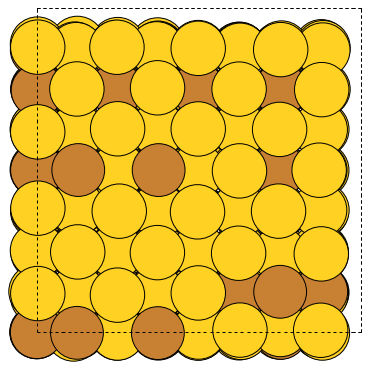

Supplement: Supplementary file 4 — Supplementary Data 1 [file 41467_2022_30687_MOESM4_ESM.zip › scripts/3_od_transition/OD-transition/CuAu3/CuAu3_4x4x4_00400K.png]

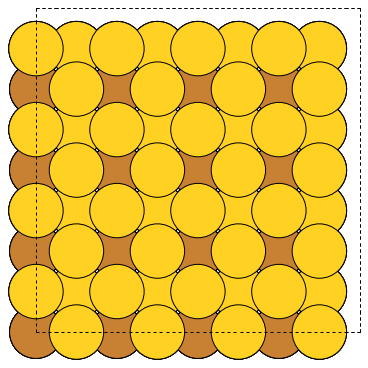

Supplement: Supplementary file 4 — Supplementary Data 1 [file 41467_2022_30687_MOESM4_ESM.zip › scripts/3_od_transition/OD-transition/CuAu3/CuAu3_4x4x4_00300K.png]

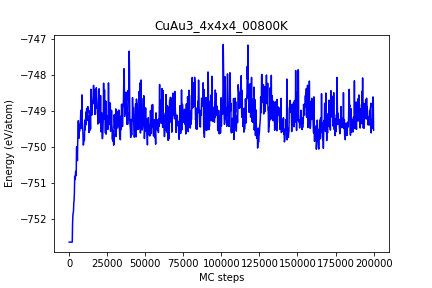

Supplement: Supplementary file 4 — Supplementary Data 1 [file 41467_2022_30687_MOESM4_ESM.zip › scripts/3_od_transition/OD-transition/CuAu3/CuAu3_4x4x4_00800K_energy.png]

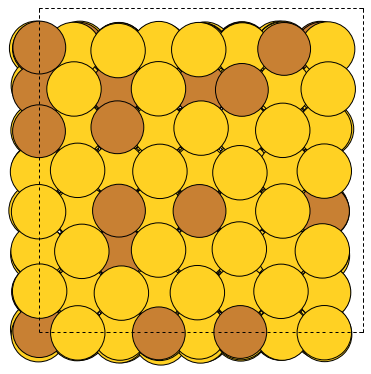

Supplement: Supplementary file 4 — Supplementary Data 1 [file 41467_2022_30687_MOESM4_ESM.zip › scripts/3_od_transition/OD-transition/CuAu3/CuAu3_4x4x4_00800K.png]

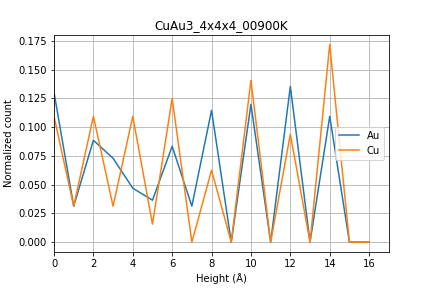

Supplement: Supplementary file 4 — Supplementary Data 1 [file 41467_2022_30687_MOESM4_ESM.zip › scripts/3_od_transition/OD-transition/CuAu3/CuAu3_4x4x4_00900K_depth_profile.png]

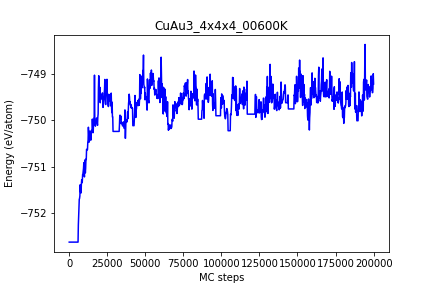

Supplement: Supplementary file 4 — Supplementary Data 1 [file 41467_2022_30687_MOESM4_ESM.zip › scripts/3_od_transition/OD-transition/CuAu3/CuAu3_4x4x4_00600K_energy.png]

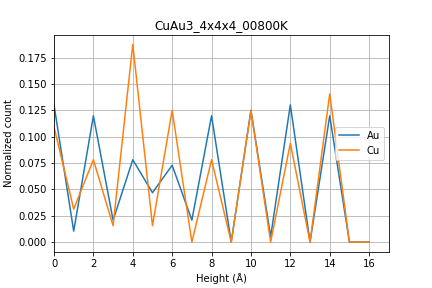

Supplement: Supplementary file 4 — Supplementary Data 1 [file 41467_2022_30687_MOESM4_ESM.zip › scripts/3_od_transition/OD-transition/CuAu3/CuAu3_4x4x4_00800K_depth_profile.png]

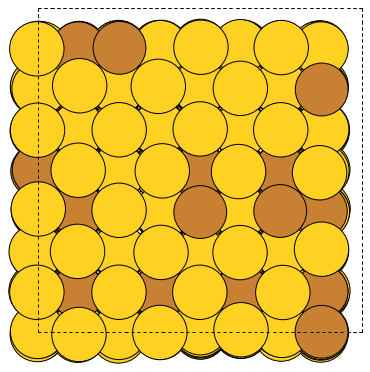

Supplement: Supplementary file 4 — Supplementary Data 1 [file 41467_2022_30687_MOESM4_ESM.zip › scripts/3_od_transition/OD-transition/CuAu3/CuAu3_4x4x4_00500K.png]

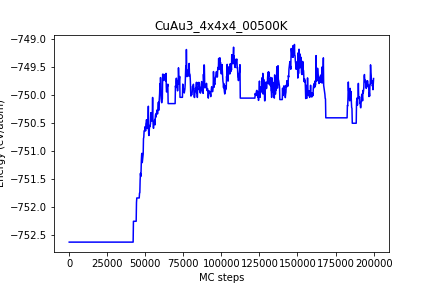

Supplement: Supplementary file 4 — Supplementary Data 1 [file 41467_2022_30687_MOESM4_ESM.zip › scripts/3_od_transition/OD-transition/CuAu3/CuAu3_4x4x4_00500K_energy.png]

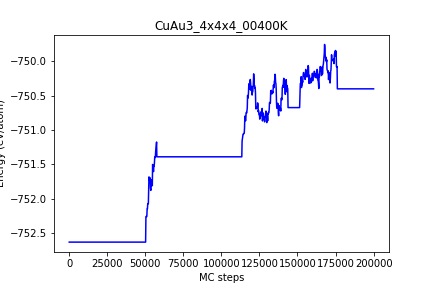

Supplement: Supplementary file 4 — Supplementary Data 1 [file 41467_2022_30687_MOESM4_ESM.zip › scripts/3_od_transition/OD-transition/CuAu3/CuAu3_4x4x4_00400K_energy.png]

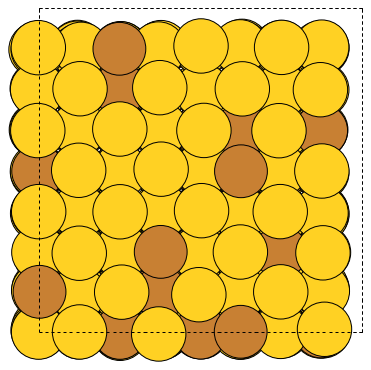

Supplement: Supplementary file 4 — Supplementary Data 1 [file 41467_2022_30687_MOESM4_ESM.zip › scripts/3_od_transition/OD-transition/CuAu3/CuAu3_4x4x4_01000K.png]

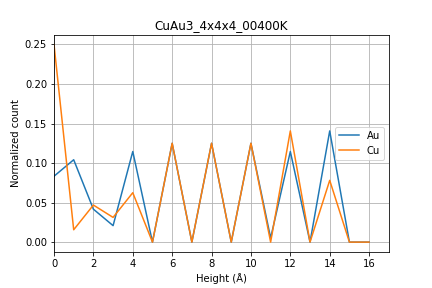

Supplement: Supplementary file 4 — Supplementary Data 1 [file 41467_2022_30687_MOESM4_ESM.zip › scripts/3_od_transition/OD-transition/CuAu3/CuAu3_4x4x4_00400K_depth_profile.png]

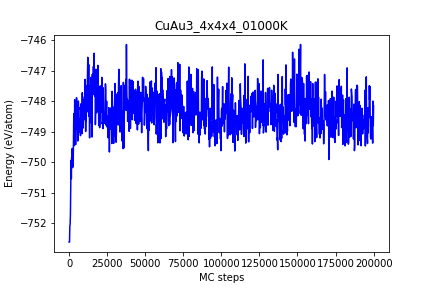

Supplement: Supplementary file 4 — Supplementary Data 1 [file 41467_2022_30687_MOESM4_ESM.zip › scripts/3_od_transition/OD-transition/CuAu3/CuAu3_4x4x4_01000K_energy.png]

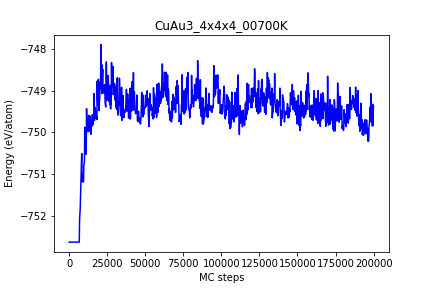

Supplement: Supplementary file 4 — Supplementary Data 1 [file 41467_2022_30687_MOESM4_ESM.zip › scripts/3_od_transition/OD-transition/CuAu3/CuAu3_4x4x4_00700K_energy.png]

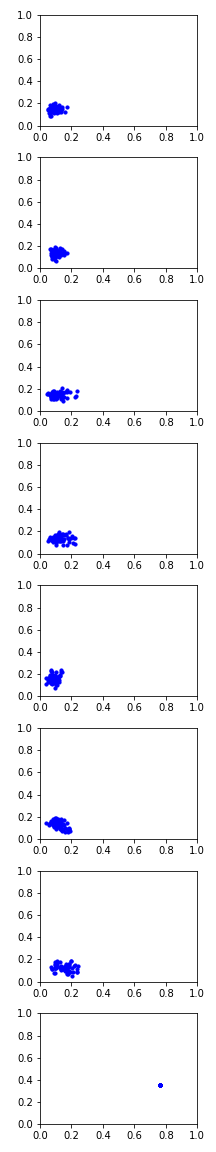

Supplement: Supplementary file 4 — Supplementary Data 1 [file 41467_2022_30687_MOESM4_ESM.zip › scripts/3_od_transition/OD-transition/CuAu3/CuAu3_Cu_q4q6.png]

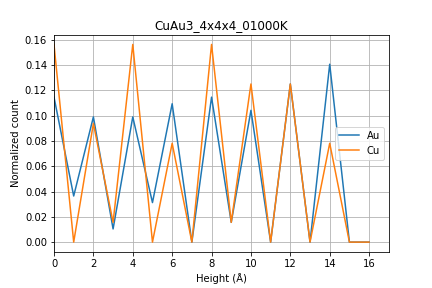

Supplement: Supplementary file 4 — Supplementary Data 1 [file 41467_2022_30687_MOESM4_ESM.zip › scripts/3_od_transition/OD-transition/CuAu3/CuAu3_4x4x4_01000K_depth_profile.png]

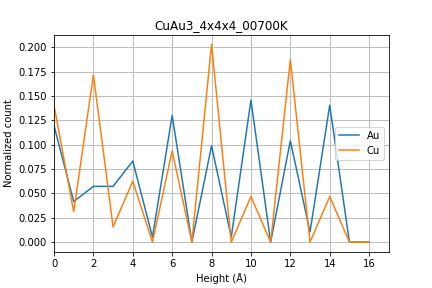

Supplement: Supplementary file 4 — Supplementary Data 1 [file 41467_2022_30687_MOESM4_ESM.zip › scripts/3_od_transition/OD-transition/CuAu3/CuAu3_4x4x4_00700K_depth_profile.png]

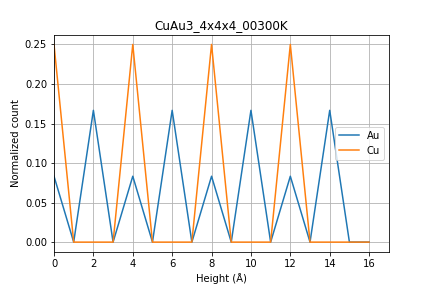

Supplement: Supplementary file 4 — Supplementary Data 1 [file 41467_2022_30687_MOESM4_ESM.zip › scripts/3_od_transition/OD-transition/CuAu3/CuAu3_4x4x4_00300K_depth_profile.png]

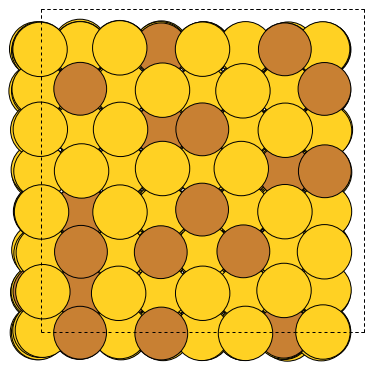

Supplement: Supplementary file 4 — Supplementary Data 1 [file 41467_2022_30687_MOESM4_ESM.zip › scripts/3_od_transition/OD-transition/CuAu3/CuAu3_4x4x4_00900K.png]

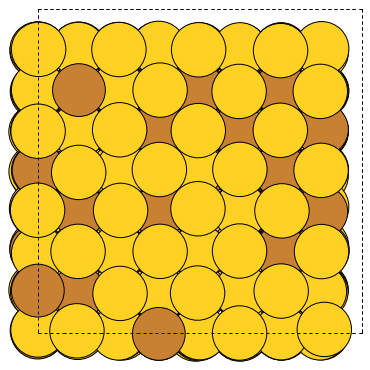

Supplement: Supplementary file 4 — Supplementary Data 1 [file 41467_2022_30687_MOESM4_ESM.zip › scripts/3_od_transition/OD-transition/CuAu3/CuAu3_4x4x4_00700K.png]

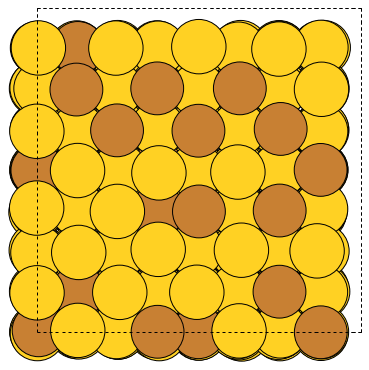

Supplement: Supplementary file 4 — Supplementary Data 1 [file 41467_2022_30687_MOESM4_ESM.zip › scripts/3_od_transition/OD-transition/CuAu3/CuAu3_4x4x4_00600K.png]

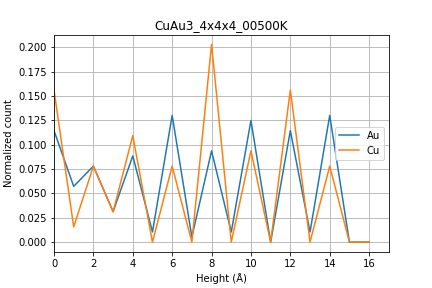

Supplement: Supplementary file 4 — Supplementary Data 1 [file 41467_2022_30687_MOESM4_ESM.zip › scripts/3_od_transition/OD-transition/CuAu3/CuAu3_4x4x4_00500K_depth_profile.png]
