# Supplementary material for: Towards universal neural network potential for material discovery applicable to arbitrary combination of 45 elements
Source: Nat Commun. 2022 May 30;13:2991. doi: 10.1038/s41467-022-30687-9 (PMC9151783; doi:10.1038/s41467-022-30687-9)
Supplement: Supplementary file 4 — Supplementary Data 1 [file 41467_2022_30687_MOESM4_ESM.zip › scripts/1_LiFeSO4F/md/fig/li_arrhenius.pdf]

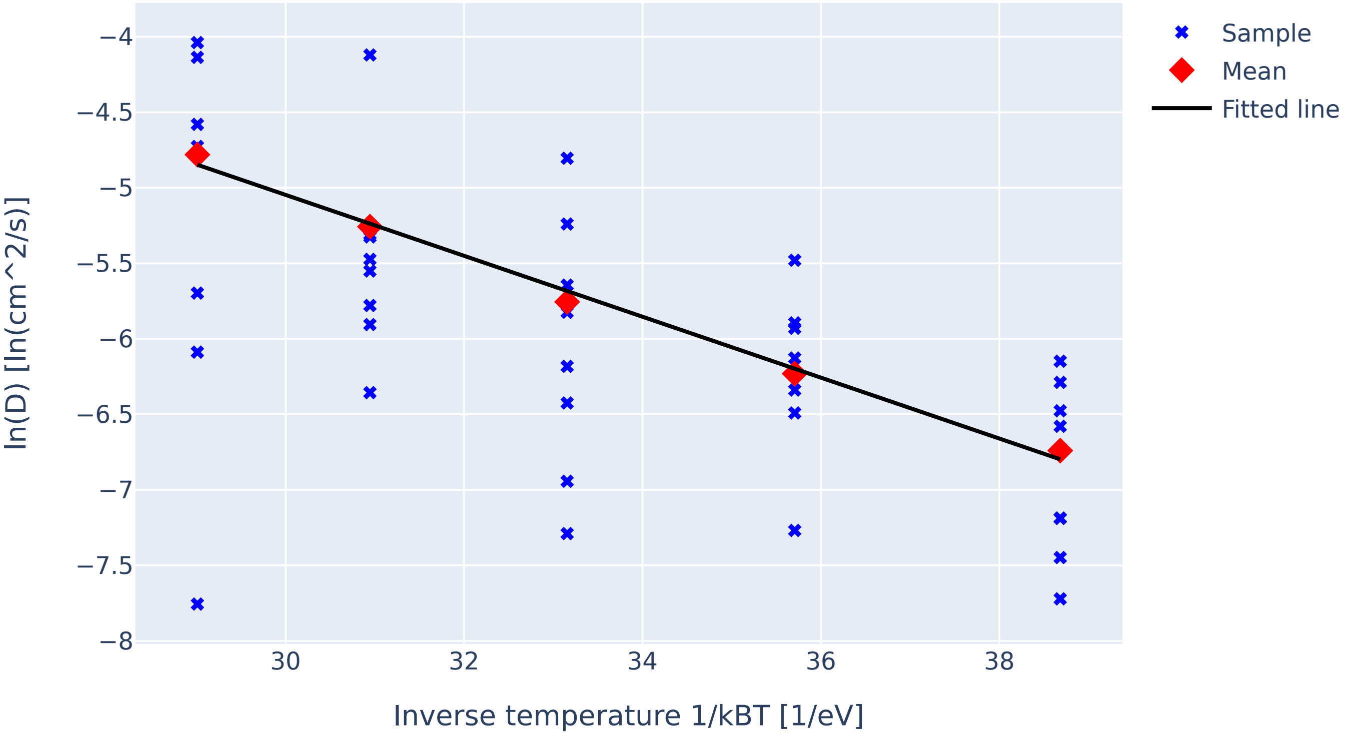


Loading [MathJax]/extensions/MathMenu.js
